# Antagonistic roles in fetal development and adult physiology for the oppositely imprinted *Grb10* and *Dlk1* genes

**DOI:** 10.1186/s12915-014-0099-8

**Published:** 2014-12-31

**Authors:** Marta Madon-Simon, Michael Cowley, Alastair S Garfield, Kim Moorwood, Steven R Bauer, Andrew Ward

**Affiliations:** Department of Biology & Biochemistry and Centre for Regenerative Medicine, University of Bath, Building 4 South, Claverton Down, Bath, BA2 7AY UK; Division of Cellular and Gene Therapies, Cellular and Tissue Therapies Branch, Food and Drug Administration, Center for Biologics Evaluation and Research, 10903 New Hampshire Avenue, Silver Spring, MD 20993-0002 USA; Present address: Department of Cell Biology, University of Geneva, CH-1211 Geneva 4, Switzerland; Department of Biological Sciences and Center for Human Health and the Environment, North Carolina State University, Raleigh, NC 27695 USA; Present address: Centre for Integrative Physiology, University of Edinburgh, Edinburgh, UK

**Keywords:** Adiposity, Body proportions, Genomic imprinting, Glucose-regulated metabolism, Growth, Mouse genetics

## Abstract

**Background:**

Despite being a fundamental biological problem the control of body size and proportions during development remains poorly understood, although it is accepted that the insulin-like growth factor (IGF) pathway has a central role in growth regulation, probably in all animals. The involvement of imprinted genes has also attracted much attention, not least because two of the earliest discovered were shown to be oppositely imprinted and antagonistic in their regulation of growth. The *Igf2* gene encodes a paternally expressed ligand that promotes growth, while maternally expressed *Igf2r* encodes a cell surface receptor that restricts growth by sequestering Igf2 and targeting it for lysosomal degradation. There are now over 150 imprinted genes known in mammals, but no other clear examples of antagonistic gene pairs have been identified. The *delta-like 1* gene (*Dlk1*) encodes a putative ligand that promotes fetal growth and in adults restricts adipose deposition. Conversely, *Grb10* encodes an intracellular signalling adaptor protein that, when expressed from the maternal allele, acts to restrict fetal growth and is permissive for adipose deposition in adulthood.

**Results:**

Here, using knockout mice, we present genetic and physiological evidence that these two factors exert their opposite effects on growth and physiology through a common signalling pathway. The major effects are on body size (particularly growth during early life), lean:adipose proportions, glucose regulated metabolism and lipid storage in the liver. A biochemical pathway linking the two cell signalling factors remains to be defined.

**Conclusions:**

We propose that *Dlk1* and *Grb10* define a mammalian growth axis that is separate from the IGF pathway, yet also features an antagonistic imprinted gene pair.

**Electronic supplementary material:**

The online version of this article (doi:10.1186/s12915-014-0099-8) contains supplementary material, which is available to authorized users.

## Background

Growth during the development of multicellular organisms requires an expansion in cell number, generally accompanied by cellular specialisation, and the formation of distinct tissues and organs. There is a wealth of information about the underlying cellular processes, including the regulation of cell survival, proliferation and differentiation, but the control of growth and proportions at the level of tissues, organs and body size remains poorly understood [1–5]. The insulin-like growth factors (IGFs), Igf1 and Igf2, promote cell survival and proliferation, and mouse knockout studies show that they have a central role in controlling body size during development [6–8]. An analogous insulin/IGF growth-regulatory pathway has been demonstrated in a number of animals, including invertebrates such as *Drosophila* [9], and may exist in all animals. In mammals two genes encoding key components of the IGF pathway, *Igf2* and *Igf2r*, are subject to regulation by genomic imprinting [10,11].

Imprinted genes have been the focus of much attention because they defy one of the central assumptions of Mendelian inheritance, that the two parental genomes can be regarded as equal. Genes regulated by genomic imprinting are expressed exclusively, or predominantly, from one parental allele in at least some of the sites where they are active [12]. The pattern of allelic expression is a characteristic feature, with some imprinted genes expressed from the paternally-inherited allele, such as the genes encoding insulin-like growth factor 2 (*Igf2*) [11] and delta-like 1 (*Dlk1*) [13], and others expressed from the maternally-inherited allele, such as those encoding the insulin-like growth factor type 2 receptor (*Igf2r*) [10] and the non-coding RNA *H19* [14,15]. The growth factor receptor bound protein 10 gene (*Grb10*) exhibits expression from the maternal allele that is widespread in fetal tissues outside of the central nervous system (CNS), but also exhibits expression from the paternal allele within the CNS [16,17]. Opposite imprinting of a single gene that expresses the same products in different tissues is currently unique to *Grb10*.

Several theories have been proposed for the evolution of imprinted genes, the most widely accepted being the parental conflict hypothesis, which was put forward before any of the imprinted genes were identified [18,19]. It holds that paternally expressed genes will function to promote offspring growth, at the expense of maternal resources, while maternally expressed genes will limit offspring growth to provide a more even distribution of resources amongst offspring throughout the mother’s reproductive lifespan. This works for species in which offspring have access to maternal resources during at least some of their early growth period, and in which females are likely to reproduce with more than one male. Thus, the theory was proposed to apply to mammals and flowering plants, in which maternal resources are made available to growing offspring through the placenta and endosperm, respectively. The species distribution remains true, with imprinted genes identified in both mammals, (including marsupials) and angiosperms [20].

Paternally expressed *Igf2* and maternally expressed *Igf2r* were two of the first imprinted genes to be identified [10,11]. Mouse knockout studies revealed a role for *Igf2* in promoting fetal growth [6] and for *Igf2r* as an inhibitor of fetal growth [21,22]. This matched perfectly with the predictions of the parental conflict hypothesis and was made more compelling by the discovery that the Igf2r, also known as the cation-independent mannose 6-phosphate receptor (CI-MPR), acts by targeting Igf2 for lysosomal degradation [22,23]. Indeed, the mammalian CI-MPR has a specific binding site for Igf2 that is not present in non-mammalian vertebrates, consistent with the Igf2r function having evolved together with genomic imprinting [24,25]. The oppositely imprinted *Igf2* and *Igf2r* genes are therefore functionally antagonistic, acting within the same biochemical pathway to regulate growth.

Over 150 imprinted genes have now been identified in placental mammals [26], a significant proportion having growth regulatory roles consistent with the parental conflict hypothesis [27,28]. It should be noted that other imprinted genes have diverse functions, notably in energy homeostasis [27,28], or brain function and behaviour [29]. Some of these functions are difficult to reconcile with the conflict hypothesis, particularly those affecting only post-natal aspects of physiology or behaviour [30], leading to the proposal of alternatives such as coadaptive evolution [31], which are not necessarily mutually exclusive with the parental conflict hypothesis [32,33]. Since the discovery of the relationship between *Igf2* and *Igf2r* there have been no other clear examples of imprinted genes with antagonistic growth functions, although oppositely imprinted transcripts at the *Gnas* locus have antagonistic roles in behaviour and physiology [34]. There are also examples of changes in one imprinted gene affecting the expression of others, potentially in a network of growth regulatory imprinted genes that includes *Igf2*, *Cdkn1c* (*p57*^*KIP2*^) and *Dlk1* [35]. Most notably, both *IGF2* and the maternally expressed cell cycle inhibitor *CDKN1C* can contribute to the overgrowth disorders seen in Beckwith-Wiedemann syndrome [36] and there is evidence that changes in *Igf2* expression can influence expression of *Cdkn1c* [37].

We have previously shown that *Grb10* has multiple roles. The maternal *Grb10* allele acts as an inhibitor of both fetal and placental growth, with mice inheriting a null allele of *Grb10* through the maternal line (*Grb10*^m/+^) being born approximately 25% to 40% larger than their wild type littermates [17,38–41]. As adults *Grb10*^*m/+*^animals are lean, with increased muscle mass, elevated insulin signalling and an enhanced ability to clear a glucose load [39,42–44]. Furthermore, expression of the maternal *Grb10* alleles in both mother and offspring have complementary roles that appear to be required for optimal offspring growth and body proportions [41]. Mice with the paternally-inherited allele of *Grb10* knocked out (*Grb10*^+/p^) exhibit normal growth and physiology, but as adults display increased social dominance, consistent with CNS-specific expression from the paternal *Grb10* allele [17]. Grb10 is an adaptor protein capable of interacting with numerous intracellular signalling molecules, notably including receptor tyrosine kinases and mammalian target of rapamycin (mTOR) [45–48]. Biochemical interactions of Grb10 with the insulin receptor (Insr) and with Igf1r are well established [45]. While there is good evidence that Grb10 acts as an inhibitor of insulin signalling in some contexts *in vivo* [39,42,43], mouse genetic experiments indicate that *Grb10* influences fetal and placental growth through a pathway independent of either *Igf2* [38], *Igf1r* or *Insr* (unpublished data). The signalling pathway through which Grb10 influences growth is currently unknown.

In contrast to the *Grb10*^*m/+*^ knockout phenotype, mice inheriting a knockout of the paternal *Dlk1* allele (*Dlk1*^*+/p*^) display significant growth retardation on the day of birth, and as adults are predisposed to accumulate excess adipose [49]. Transgenic mice overexpressing *Dlk1* from an adipose-specific promoter exhibit reduced adipose mass, impaired glucose tolerance and decreased insulin resistance [50,51]. The adiposity phenotypes of the *Dlk1* knockout and transgenic mice are consistent with abundant evidence that Dlk1, also known as preadipocyte factor-1 (pref-1), is an important regulator of adipogenesis [52]. Enhanced fetal growth of *Grb10*^*m/+*^ mice is associated with expansion of the labyrinthine portion of the placenta [40], whereas placental labyrinth volume is reduced in *Dlk1*^*+/p*^ mice [53]. As adults, *Grb10*^*m/+*^ mice have increased muscle mass, due to an increase in myofiber number [44,54], while *Dlkl*^*−/−*^ animals exhibit reduced muscle mass associated with a delay in myogenesis during fetal development [55]. Here, we test the hypothesis that the oppositely imprinted *Grb10* and *Dlk1* genes have antagonistic roles in a common genetic pathway. Analyses of growth and physiological phenotypes of *Grb10*^*m/+*^*/Dlk1*^*+/p*^ double knockout animals support this hypothesis. Our data indicate that, in addition to the IGF pathway, a second mammalian fetal growth regulatory axis exists in which oppositely imprinted genes have evolved roles consistent with the parental conflict hypothesis.

## Results

### *Grb10*^*m/+*^*/Dlk1*^*+/p*^ double knockout mice have a fetal overgrowth phenotype similar to that of *Grb10*^*m/+*^ single knockout mice, including increased cell proliferation along with altered lengths of S- and G2-phases of the cell cycle

**Figure 1:**
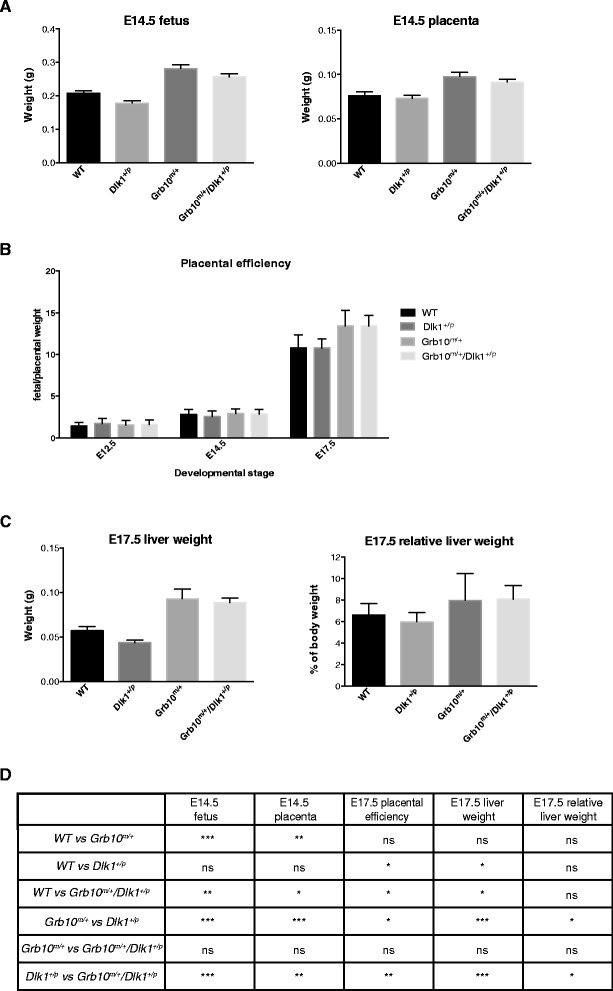
**Analyses of fetal, placental and liver weights during mid- to late-gestation. A)** At E14.5 *Grb10* ^*m/+*^ and *Grb10* ^*m/+*^ */Dlk1* ^*+/p*^ fetuses and placentae were significantly overgrown when compared to wild type and *Dlk1* ^*+/p*^ fetuses. **B)** Placental efficiency, calculated as a ratio of fetal/placental mass, was significantly increased at E17.5, but not E12.5 or E14.5, for *Grb10* ^*m/+*^ and *Grb10* ^*m/+*^ */Dlk1* ^*+/p*^ conceptuses when compared to both wild type and *Dlk1* ^*+/p*^. **C)** At E17.5 *Grb10* ^*m/+*^ and *Grb10* ^*m/+*^ */Dlk1* ^*+/p*^ fetal livers were significantly heavier than both wild type and *Dlk1* ^*+/p*^ livers. When expressed as a proportion of body weight (relative weights) *Grb10* ^*m/+*^ */Dlk1* ^*+/p*^ fetal livers were significantly enlarged compared to wild type and *Dlk1* ^*+/p*^ livers. **D)** Table summarising results of statistical analyses in A-C. All values represent means ± SEM, one way ANOVA with Tukey’s *post-hoc* analysis. For E12.5 WT n = 23, *Dlk1* ^*+/p*^ n = 13, *Grb10* ^*m/+*^ n = 13, *Grb10* ^*m/+*^ */Dlk1* ^*+/p*^ n = 16 ; for E14.5 WT n = 19, *Dlk1* ^*+/p*^ n = 22, *Grb10* ^*m/+*^ n = 22, *Grb10* ^*m/+*^ */Dlk1* ^*+/p*^ n = 25; for E17.5 WT n = 4, *Dlk1* ^*+/p*^ n = 8, *Grb10* ^*m/+*^ n = 6 *Grb10* ^*m/+*^ */Dlk1* ^*+/p*^ n = 8; * *P* <0.05; ** *P* <0.01; *** *P* <0.001. ANOVA, analysis of variance; E, embryonic day; SEM, standard error of the mean; vs, versus; WT, wild type.

To investigate growth phenotypes resulting from ablation of the *Grb10* and *Dlk1* genes, fetal and placental wet weights together with placental efficiencies were analysed at different gestational stages (Figure 1 and Additional file 1: Figure S1). At embryonic day (E)12.5 *Grb10*^*m/+*^and *Grb10*^*m/+*^*/Dlk1*^*+/p*^ fetuses were significantly heavier than wild type and also *Dlk1*^*+/p*^ littermate controls, with no statistically significant differences seen in placental mass (see Additional file 1: Figure S1A,C). Both *Grb10*^*m/+*^ and *Grb10*^*m/+*^*/Dlk1*^*+/p*^ fetuses and placentae were significantly enlarged at E14.5, again in comparison with both wild type and *Dlk1*^*+/p*^ controls (Figure 1A,D). The same trend was observed at E17.5, with both *Grb10*^*m/+*^and *Grb10*^*m/+*^*/Dlk1*^*+/p*^ fetuses exhibiting significant overgrowth when compared to wild type and *Dlk1*^*+/p*^ fetuses, but in the case of placentae statistically significant overgrowth was observed only in comparison to *Dlk1*^*+/p*^ littermates (see Additional file 1: Figure S1B,C). Determination of fetal/placental weight ratios as a measure of placental efficiency revealed significantly improved efficiencies for both *Grb10*^*m/+*^and *Grb10*^*m/+*^*/Dlk1*^*+/p*^ placentae at E17.5 but not at E12.5 or E14.5 (Figure 1B,D). We previously found that *Grb10*^*m/+*^ neonates (on the day of birth) exhibited disproportionate liver enlargement [17,38], and here we identified significant overgrowth in wet weights and relative weights of both *Grb10*^*m/+*^and *Grb10*^*m/+*^*/Dlk1*^*+/p*^ E17.5 livers (Figure 1C,D).

**Figure 2:**
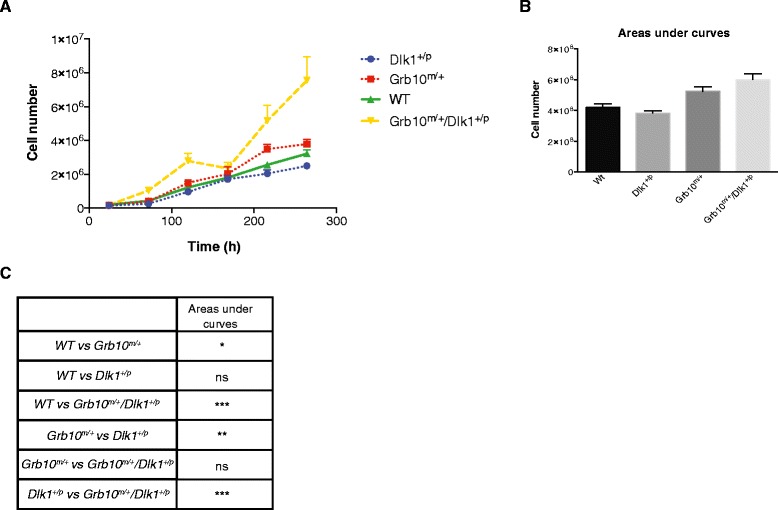
**Proliferation rates of E14.5 primary embryonic fibroblasts (PMEFs). A)** Growth curves were plotted for E14.5 PMEFs seeded at equivalent densities and then cultured for 264 hours. Each data point represents the mean of three independent experiments (each comparing cells of all four genotypes), four replicates for each time point, counted twice. **B)** Areas under curves calculated using data from A. **C)** Statistical analysis of total areas under growth curves revealed that *Grb10* ^*m/+*^ and *Grb10* ^*m/+*^ */Dlk1* ^*+/p*^ PMEFs each proliferated significantly more than both wild type and *Dlk1* ^*+/p*^ cells. All values represent means ± SEM, tested using one way ANOVA with Tukey’s *post-hoc* analysis; * *P* <0.05; ** *P* <0.01; *** *P* <0.001. For each genotype, n = 3. ANOVA, analysis of variance; E, embryonic day; SEM, standard error of the mean.

The tissue overgrowth observed from E12.5 onwards in both *Grb10*^*m/+*^and *Grb10*^*m/+*^*/Dlk1*^*+/p*^ fetuses might be associated with either, or both, increased cell size and cell number. To investigate the cellular basis of tissue overgrowth we first analysed proliferation rates of primary mouse embryonic fibroblasts (PMEFs) derived from E14.5 fetuses. PMEFs were seeded at the same initial density and monitored over a period of 11 days, revealing that *Grb10*^*m/+*^and *Grb10*^*m/+*^*/Dlk1*^*+/p*^ cells were hyperproliferative (Figure 2A). Note that at 24 hours, the first time-point cells were counted after initial seeding, there was essentially no difference in cell numbers. Statistical analysis of areas under the growth curves showed that accumulation of PMEFs derived from both *Grb10*^*m/+*^and *Grb10*^*m/+*^*/Dlk1*^*+/p*^ fetuses was significantly increased in comparison with those derived from wild type and *Dlk1*^*+/p*^ fetuses (Figure 2B,C). No statistically significant deviation from wild type growth was shown by *Dlk1*^*+/p*^ cells, although they did exhibit a consistent trend to proliferate at a slightly lower rate than wild type cells.

**Figure 3:**
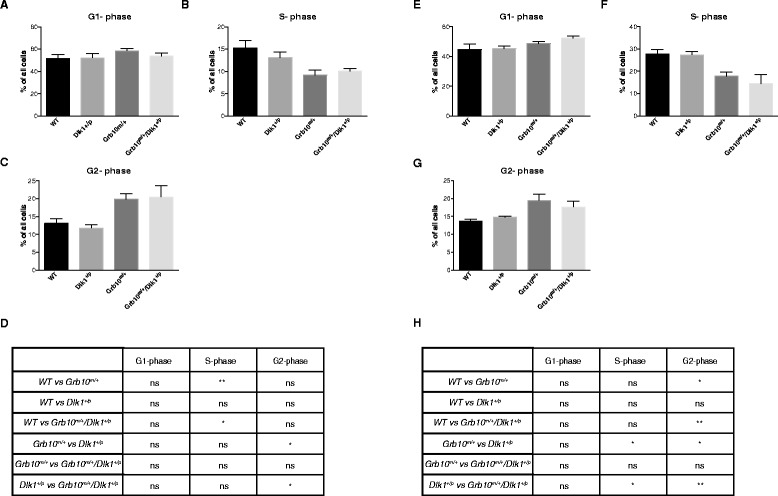
**FACS analysis of cell cycle fractions in propidium iodide stained cultured E14.5 PMEFs and disaggregated E11.5 fetuses. A-C)** Relative DNA content of passage 3 PMEFs, estimated for 100,000 cells per sample. Cells were allocated to bins representing the G1- **(A)**, S- **(B)** and G2- **(C)** phases of the cell cycle. Cell populations that were <2n and >4n were excluded from the analysis. **D)** Table summarising results of statistical analysis of data in A-C. All values represent means ± SEM, one way ANOVA with Tukey’s *post-hoc* analysis. WT n = 6, *Dlk1* ^*+/p*^ n = 5, *Grb10* ^*m/+*^ n = 7, *Grb10* ^*m/+*^ */Dlk1* ^*+/p*^ n = 6; **E-G)** Cells derived directly from wild type, *Dlk1* ^*+/p*^, *Grb10* ^*m/+*^ and *Grb10* ^*m/+*^ */Dlk1* ^*+/p*^ E11.5 fetuses were used to estimate relative DNA content for 100,000 cells per sample. Cells were allocated to bins representing the G1- **(E)**, S- **(F)** and G2- **(G)** phases of the cell cycle. **H)** Table summarising results of statistical analysis of data in **E**-**G**. All values represent means ± SEM, one way ANOVA with Tukey’s *post-hoc* analysis. WT n = 7, *Dlk1* ^*+/p*^ n = 8, *Grb10* ^*m/+*^ n = 6, *Grb10* ^*m/+*^ */Dlk1* ^*+/p*^ n = 5. For both PMEFs **(A-D)** and E11.5 fetal cells **(E-H)** significantly lower percentages of both *Grb10* ^*m/+*^ and *Grb10* ^*m/+*^ */Dlk1* ^*+/p*^ cells were found in S-phase of the cell cycle in comparison to either wild type (PMEFs) or *Dlk1* ^*+/p*^ (E11.5 embryo) cells. Conversely, significantly higher percentages of both *Grb10* ^*m/+*^ and *Grb10* ^*m/+*^ */Dlk1* ^*+/p*^ cells were found in G2-phase of the cell cycle in comparison to wild type and *Dlk1* ^*+/p-*^ E11.5 embryo cells. Similar trends were seen for PMEFs but only the comparisons of *Grb10* ^*m/+*^ and *Grb10* ^*m/+*^ */Dlk1* ^*+/p*^ with wild type reached significance. No significant differences were observed for numbers of cells in G1 for comparisons between any of the four genotypes; * *P* <0.05. ANOVA, analysis of variance; E, embryonic day; FACS, fluorescence activated cell sorting; PMEF, primary mouse embryonic fibroblasts; SEM, standard error of the mean; WT, wild type.

Fluorescence activated cell sorting (FACS) was employed to examine the basis for the hyperproliferative phenotype of *Grb10*^*m/+*^and *Grb10*^*m/+*^*/Dlk1*^*+/p*^ E14.5 PMEFs and to investigate the possibility of cellular hypertrophy. No deviations from wild type were found in the ranges of cell sizes upon FACS analysis of *Dlk1*^*+/p*^, *Grb10*^*m/+*^and *Grb10*^*m/+*^*/Dlk1*^*+/p*^ cells from E14.5 fetuses, suggesting that the observed overgrowth phenotypes in *Grb10*^*m/+*^and *Grb10*^*m/+*^*/Dlk1*^*+/p*^ fetuses were not the result of cell hypertrophy (see Additional file 2: Figure S2A,B). In contrast, analysis of cell cycle stages revealed statistically significant increases in the proportion of *Grb10*^*m/+*^and *Grb10*^*m/+*^*/Dlk1*^*+/p*^ cells in G2-phase compared to *Dlk1*^*+/p*^ cells, along with decreased numbers of *Grb10*^*m/+*^and *Grb10*^*m/+*^*/Dlk1*^*+/p*^ cells in S-phase compared to wild type (Figure 3A-D). To eliminate potential confounds associated with cell culture, FACS analysis was also undertaken using freshly derived single cell suspensions from E11.5 fetuses. We again found no changes in cell size distribution (see Additional file 2: Figure S2C,D). Also consistent with data from PMEFs, *Grb10*^*m/+*^and *Grb10*^*m/+*^*/Dlk1*^*+/p-*^ fetuses showed significantly higher percentages of cells in G2-phase of the cell cycle, compared to both wild type and *Dlk1*^*+/p-*^ fetuses, with decreased numbers in S-phase, although this time statistically significant only in comparison to *Dlk1*^*+/p*^ fetuses (Figure 3E-H).

### At birth and as adults *Grb10*^*m/+*^and *Grb10*^*m/+*^*/Dlk1*^*+/p*^ mice share characteristic changes in body weight and proportions that distinguish them from wild type and *Dlk1*^*+/p*^ animals

**Figure 4:**
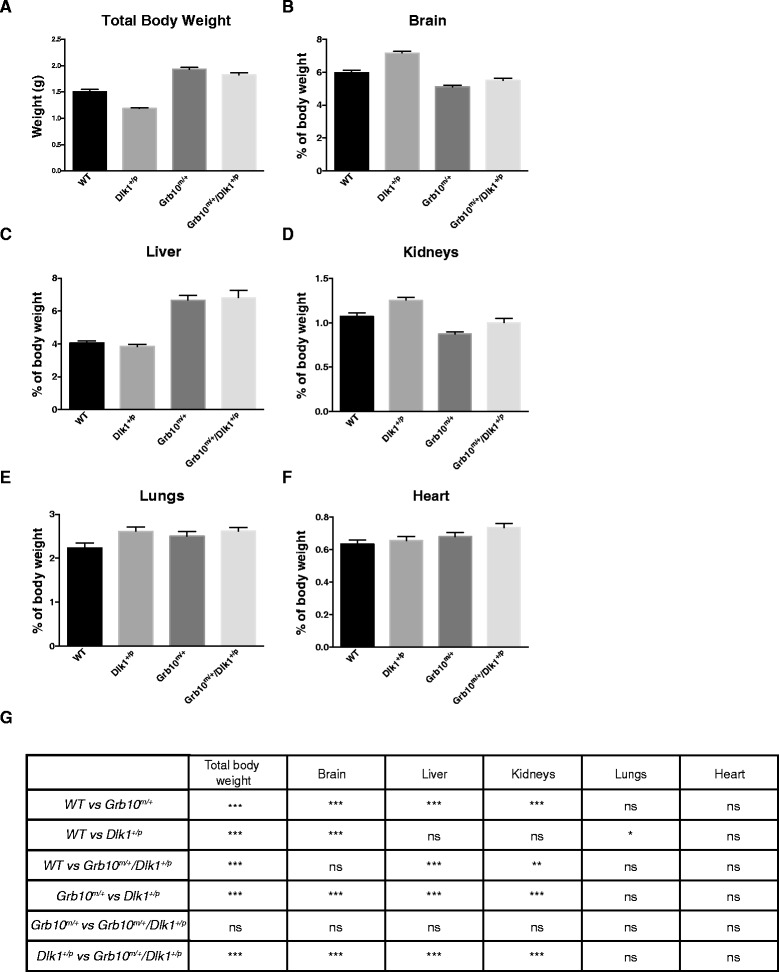
**Whole body and relative organ weight analysis of neonates. A)** *Dlk1* ^*+/p*^ mice were significantly growth retarded whereas *Grb10* ^*m/+*^ and *Grb10* ^*m/+*^ */Dlk1* ^*+/p*^ animals demonstrated whole body overgrowth when compared to wild type and *Dlk1* ^*+/p*^ mice. Note, this graph is the same as that shown in Additional file 3: Figure S3A, reproduced here for convenience. **B)** Cranial sparing was observed, such that when brain weights were expressed as a percentage of total body weight *Dlk1* ^*+/p*^ mice had proportionately enlarged brains in comparison to mice of all other genotypes. Conversely, the brains of *Grb10* ^*m/+*^ and *Grb10* ^*m/+*^ */Dlk1* ^*+/p*^ animals were proportionately reduced in size compared to wild type and *Dlk1* ^*+/p*^ mice (though note, not significantly so in the case of *Grb10* ^*m/+*^ */Dlk1* ^*+/p*^ versus wild type animals). **C)** *Grb10* ^*m/+*^ and *Grb10* ^*m/+*^ */Dlk1* ^*+/p*^ mice had disproportionally overgrown livers when compared to wild type and *Dlk1* ^*+/p*^ animals. **D)** Kidney sparing was seen in both *Grb10* ^*m/+*^ and *Grb10* ^*m/+*^ */Dlk1* ^*+/p*^ animals compared to wild type and *Dlk1* ^*+/p*^ mice. Conversely, kidney weight was proportionately increased in *Dlk1* ^*+/p*^ animals compared with mice of all other genotypes (though note, not significantly so in the case of *Dlk1* ^*+/p*^ versus wild type animals). **E)** Overgrowth of lungs was noted in *Dlk1* ^*+/p*^ mice compared to wild type mice. **F)** Proportionate growth of hearts was seen in all the analysed genotypes. **G)** Table summarising results of statistical analysis. All values represent means ± SEM, analysed using one way ANOVA with Tukey’s *post-hoc* analysis. WT n = 19, *Dlk1* ^*+/p*^ n = 36, *Grb10* ^*m/+*^ n = 23, *Grb10* ^*m/+*^ */Dlk1* ^*+/p*^ n = 22; * *P* <0.05; ** *P* <0.01; *** *P* <0.001. ANOVA, analysis of variance; SEM, standard error of the mean; WT, wild type.

We next analysed body weight, proportions and selected histological features of mice at post-natal stages from the day of birth (neonates) to nine months of age. As expected, neonatal *Dlk1*^*+/p*^ knockout mice were significantly smaller than wild type mice whilst *Grb10*^*m/+*^ knockout mice were significantly overgrown (Figure 4A,G). *Grb10*^*m/+*^*/Dlk1*^*+/p*^ double knockout neonates also displayed an overgrowth phenotype, being significantly heavier than wild type and *Dlk1*^*+/p*^ animals but not significantly different from *Grb10*^*m/+*^ single knockouts. Analysis of organ wet weights showed that liver, lung and heart were each significantly enlarged in both *Grb10*^*m/+*^and *Grb10*^*m/+*^*/Dlk1*^*+/p*^ double knockout neonates (see Additional file 3: Figure S3), and when expressed as a proportion of body weight, heart (Figure 4F,G) and lung (Figure 4E,G) were essentially in proportion with total body weight, with liver disproportionately enlarged (Figure 4C,G), as previously shown for *Grb10*^*m/+*^ mice [17,38]. The wet weights of brain and kidney exhibited no significant differences between the genotypes (see Additional file 3: Figure S3) and consequently, as a proportion of body weight, appeared enlarged in *Dlk1*^*+/p*^ neonates and reduced in *Grb10*^*m/+*^ neonates (Figure 4B,D,G). In *Grb10*^*m/+*^*/Dlk1*^*+/p*^ double knockout neonates relative weights of both brain and kidney were significantly different to those of *Dlk1*^*+/p*^ but not wild type or *Grb10*^*m/+*^ animals. Lungs of *Dlk1*^*+/p*^ animals were significantly enlarged in comparison to wild type (Figure 4E,G).

**Figure 5:**
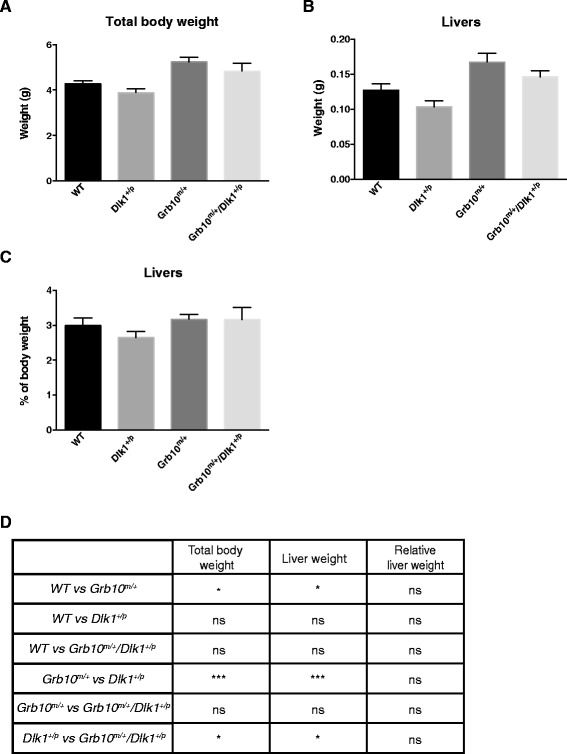
**Whole body, liver wet weight and relative liver weight analysis of one-week-old mice. A)** Significantly increased body weights were noted in *Grb10* ^*m/+*^ compared to wild type and *Dlk1* ^*+/p*^ and in *Grb10* ^*m/+*^ */Dlk1* ^*+/p*^ compared to *Dlk1* ^*+/p*^ mice whereas weights of *Dlk1* ^*+/p*^ animals did not differ from wild types. **B)** Analysis of wet weights of the livers revealed significant enlargement in *Grb10* ^*m/+*^ compared to wild type and *Dlk1* ^*+/p*^ mice and in *Grb10* ^*m/+*^ */Dlk1* ^*+/p*^ compared to *Dlk1* ^*+/p*^ animals. **C)** No differences were found in relative liver weights. **D)** Table summarising results of statistical analysis. All values represent means ± SEM, analysed using one way ANOVA with Tukey’s *post-hoc* analysis. WT n = 10, *Dlk1* ^*+/p*^ n = 9, *Grb10* ^*m/+*^ n = 15, *Grb10* ^*m/+*^ */Dlk1* ^*+/p*^ n = 8; * *P* <0.05; *** *P* <0.001. ANOVA, analysis of variance; SEM, standard error of the mean; WT, wild type.

To investigate if the identified body weight differences detected at birth would persist in postnatal life we next analysed wild type, *Grb10*^*m/+*^, *Dlk1*^*+/p*^ and *Grb10*^*m/+*^*/Dlk1*^*+/p*^ mice at one week, three to six months and more than six to nine months of age. At one week, persistent overgrowth was seen in *Grb10*^*m/+*^ (compared to wild type and *Dlk1*^*+/p*^) and *Grb10*^*m/+*^*/Dlk1*^*+/p*^ (compared to *Dlk1*^*+/p*^) knockout mice (Figure 5A,D). Wet weights of the *Grb10*^*m/+*^and *Grb10*^*m/+*^*/Dlk1*^*+/p*^ livers were significantly elevated at this stage (Figure 5B,D). However, when liver weights were expressed as percentages of the total body weights, no significant differences between the genotypes were found, indicating a shift from the disproportionate overgrowth seen at birth to a proportional increase in liver weight in *Grb10*^*m/+*^and *Grb10*^*m/+*^*/Dlk1*^*+/p*^ mice (Figure 5C,D). At three to six and more than six to nine months of age, body weights were again compared, along with wet weights and relative weights of selected organs (data summarised in Table 1). Only males three- to six-months old showed a significant difference in body weight, with *Grb10*^*m/+*^ being heavier than wild type and *Dlk1*^*+/p*^ animals. Several of the analysed organs showed significant differences in weights between genotypes and there was a notable tendency for *Grb10*^*m/+*^and *Grb10*^*m/+*^*/Dlk1*^*+/p*^ mice to group together. These differences included increased pancreas weights and decreased kidney weights in *Grb10*^*m/+*^and *Grb10*^*m/+*^*/Dlk1*^*+/p*^ mice that were consistent between males and females of the same age and/or between animals at the two different ages. The livers of *Grb10*^*m/+*^and *Grb10*^*m/+*^*/Dlk1*^*+/p*^ mice were significantly smaller than those of *Dlk1*^*+/p*^ animals in males three- to six-months old and exhibited a similar trend in the other adult cohorts.

**Table 1:** **Summary of significant changes in weight and proportions of adult mice**

**Tissues**	**3- to 6-month-old males**	**>6- to 9-month-old males**	**3- to 6-month-old females**	**>6-to 9-month-old females**
Body weight	ns	Grb10 ↑ (vs WT and Dlk1)	ns	ns
Pancreas	Grb10 andDKO ↑ (vs Dlk1)	Grb10 and DKO ↑ (vs WT & Dlk1)	ns	DKO ↑ (vs Dlk1)
Pancreas %	DKO ↑ (vs Dlk1)	DKO ↑(vs Dlk1)	ns	DKO ↑ (vs Dlk1)
		Grb10 ↑ (vs WT and Dlk1)		
Kidneys	Grb10 and DKO ↓ (vs WT)	ns	Grb10 and DKO ↓ (vs WT)	ns
Kidneys %	ns	ns	Grb10 and DKO ↓ (vs WT)	ns
Gonadal fat	ns	Dlk1 ↑ (vs DKO)	ns	Grb10 ↓ (vs WT and Dlk1)
Gonadal fat %	ns	Dlk1 ↑ (vs DKO)	ns	ns
Renal fat	ns	Dlk1 ↑ (vs WT, Grb10 and DKO)	ns	ns
Renal fat %	ns	Dlk1 ↑ (vs WT, Grb10 and DKO)	ns	ns
Liver %	Grb10 and DKO ↓ (vs DLk1)	ns	ns	ns
Testes	Dlk1 ↓ (vs WT and Grb10)	NA	Grb10 ↑ (vs WT and Dlk1)	NA

Cohorts of mice were analysed at ages 3 to 6 months and >6 to 9 months. Indicated are increases (↑) and decreases (↓) in wet weight or weight of organs expressed as a proportion of body weight (%), with comparison groups stated in brackets. Genotypes have been abbreviated such that *Grb10* ^*m/+*^ is represented as Grb10, *Dlk1* ^*+/p*^ as Dlk1 and *Grb10* ^*m/+*^ */Dlk1* ^*+/p*^ (or double knockouts) as DKO. Data were subject to one way ANOVA with Tukey’s *post-hoc* analysis and only significant differences (*P* <0.05 or less) are shown. For 3- to 6-month-old males, WT n = 7, Dlk1^+/p^ n = 6, Grb10^m/+^ n = 6, Grb10^m/+^/Dlk1^+/p^ n = 6; 3- to 6-month-old females WT n = 7, Dlk1^+/p^ n = 6, Grb10^m/+^ n = 6, Grb10^m/+^/Dlk1^+/p^ n = 7; >6- to 9-month-old males WT n = 7, Dlk1^+/p^ n = 6, Grb10^m/+^ n = 6, Grb10^m/+^/Dlk1^+/p^ n = 6; >6- to 9-month-old females WT n = 6, Dlk1^+/p^ n = 6, Grb10^m/+^ n = 6, Grb10^m/+^/Dlk1^+/p^ n = 5. ANOVA, analysis of variance; NA, not applicable; ns, no significant differences (*P* >0.05); vs, versus; WT, wild type.

### *Grb10*^*m/+*^and *Grb10*^*m/+*^*/Dlk1*^*+/p*^ mice share age-related changes in liver histology that distinguish them from wild type and *Dlk1*^*+/p*^ animals

**Figure 6:**
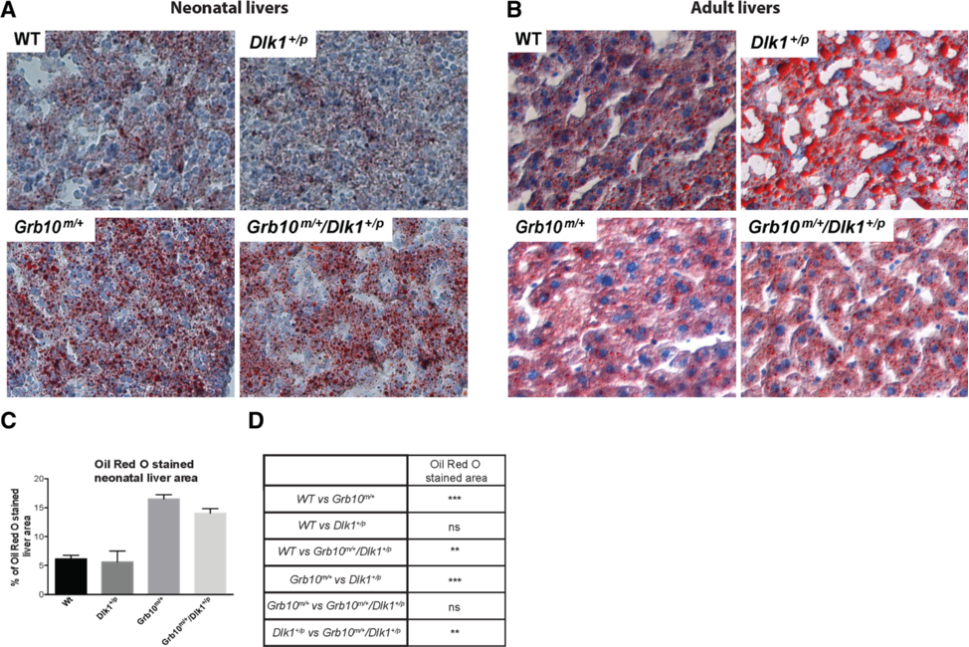
**Histology of neonatal and adult livers stained with Oil Red O. A)** An increased number of lipid droplets was observed in cryosectioned *Grb10* ^*m/+*^ and *Grb10* ^*m/+*^ */Dlk1* ^*+/p*^ neonatal livers following Oil Red O staining. **B)** An increased number of lipid droplets was observed in Oil Red O stained adult livers from *Dlk1* ^*+/p*^ knockout mice. Images presented in Panels A and B show representative sections for each of the analysed genotypes. **C)** Quantification of histological sections (from three individuals for each genotype) of neonatal liver stained with Oil red O (percentage of area stained), showing increased staining for both *Grb10* ^*m/+*^ and *Grb10* ^*m/+*^ */Dlk1* ^*+/p*^ livers. **D)** Table summarising results of statistical analysis of data in C. All values represent means ± SEM and have been subject to one way ANOVA with *post hoc* Tukey’s analysis. WT n = 3, *Dlk1* ^*+/p*^ n = 3, *Grb10* ^*m/+*^ n = 3 and *Grb10* ^*m/+*^ */Dlk1* ^*+/p*^ n = 3; ** *P* <0.01, ***P <0.001. ANOVA, analysis of variance; SEM, standard error of the mean; WT, wild type.

The differences in liver weight and proportions that we observed in *Grb10*^*m/+*^and *Grb10*^*m/+*^*/Dlk1*^*+/p*^ mice from E17.5 to adult stages were accompanied by striking changes in histology (Figure 6 and Additional file 4: Figure S4). In comparisons of tissue sections stained with haematoxylin and eosin (H & E) for all four genotypes, no obvious differences were found in E14.5 fetal liver (data not shown); however, the presence of numerous clear, round spaces was noted in neonatal *Grb10*^*m/+*^ and *Grb10*^*m/+*^*/Dlk1*^*+/p*^ livers (see Additional file 4: Figure S4A) which were also present in approximately half of the examined *Grb10*^*m/+*^ and *Grb10*^*m/+*^*/Dlk1*^*+/p*^ livers from one-week-old mice (data not shown). In contrast, *Grb10*^*m/+*^ and *Grb10*^*m/+*^*/Dlk1*^*+/p*^ livers from three-month-old male mice were essentially indistinguishable from wild type, but at this stage *Dlk1*^*+/p*^ livers were found to have an abnormal accumulation of larger clear, round spaces (see Additional file 4: Figure S4B). Oil Red O staining of cryosections revealed that *Grb10*^*m/+*^ and *Grb10*^*m/+*^*/Dlk1*^*+/p*^ neonatal livers had increased staining compared with wild type and *Dlk1*^*+/p*^ samples (Figure 6A,C,D), indicating the accumulation of lipid in a manner consistent with the appearance of clear spaces in H & E stained sections (see Additional file 4: Figure S4A). Similarly, Oil Red O staining of adult liver cryosections revealed marked lipid droplet accumulation in *Dlk1*^*+/p*^ but not in wild type, *Grb10*^*m/+*^ and *Grb10*^*m/+*^*/Dlk1*^*+/p*^ (Figure 6B), again in keeping with the observation of clear spaces in H & E stained sections (see Additional file 4: Figure S4B).

### Dual energy X-ray absorptiometry analysis of adult body composition shows both *Grb10*^*m/+*^ and *Grb10*^*m/+*^*/Dlk1*^*+/p*^ mice have increased lean tissue content, while *Dlk1*^*+/p*^ mice have a higher adipose proportion and elevated serum triglycerides

**Figure 7:**
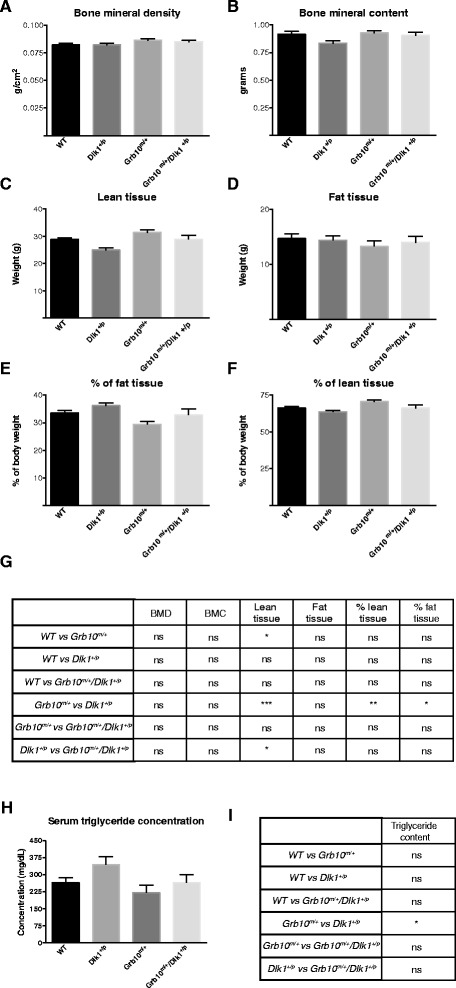
**DXA analysis of male mice and levels of triglycerides in serum.** Carcasses of male animals three- to nine-months old were subject to body composition analysis by Dual X-ray absorptiometry (DXA). No differences were seen in: **A)** bone mineral density (BMD); or **B)** bone mineral content (BMC). **C)** Total lean tissue mass was significantly elevated in *Grb10* ^*m/+*^ animals when compared to wild type and *Dlk1* ^*+/p*^ mice and in *Grb10* ^*m/+*^ */Dlk1* ^*+/p*^ animals when compared to *Dlk1* ^*+/p*^ *.* **D)** No differences were observed in total fat tissue content. **E)** Lean mass as a percentage of total body mass was significantly increased in *Grb10* ^*m/+*^ mice in comparison to *Dlk1* ^*+/p*^ mice. **F)** Fat mass as a percentage of total body mass was significantly reduced in *Grb10* ^*m/+*^ mice in comparison to *Dlk1* ^*+/p*^ mice. **G)** Table summarising results of statistical analysis of data in **A-F**. All values represent means ± SEM and have been subject to one way ANOVA with *post hoc* Tukey’s analysis. WT n = 14, *Dlk1* ^*+/p*^ =12, *Grb10* ^*m/+*^ n = 12 and *Grb10* ^*m/+*^ */Dlk1* ^*+/p*^ n = 12; * *P* <0.05; ** *P* <0.01; *** *P* <0.001. **H)** Triglyceride levels in blood serum measured in three-month-old male mice. *Dlk1* ^*+/p*^ mice were found to have significantly elevated levels of triglycerides in blood serum in comparison to *Grb10* ^*m/+*^. **I)** Table summarising results of statistical analysis of data in H. All values represent means ± SEM and have been subject to one way ANOVA with *post hoc* Tukey’s analysis. WT n = 6, *Dlk1* ^*+/p*^ n = 6, *Grb10* ^*m/+*^ n = 6 and *Grb10* ^*m/+*^ */Dlk1* ^*+/p*^ n = 6; * *P* <0.05. ANOVA, analysis of variance; SEM, standard error of the mean; WT, wild type.

To further evaluate body proportions among mice of the four different genotypes, we performed dual energy X-ray absorptiometry (DXA) analyses of adult males (Figure 7) and females (see Additional file 5: Figure S5). No significant differences in bone mineral density (BMD) and bone mineral content (BMC) were identified in male mice, indicating no major changes in bone quantity and composition (Figure 7A,B,G). Female *Dlk1*^*+/p*^ mice had significantly reduced BMC compared to wild type controls, and this was the only difference in BMC or BMD detected (see Additional file 5: Figure S5A,B,G). Lean tissue content was significantly increased for *Grb10*^*m/+*^ male mice when compared to wild type and *Dlk1*^*+/p*^ mice and also in *Grb10*^*m/+*^*/Dlk1*^*+/p*^ mice when compared to *Dlk1*^*+/p*^ (Figure 7C,G). *Grb10*^*m/+*^ female mice also had significantly higher content of lean tissue, but only when compared to *Dlk1*^*+/p*^ (see Additional file 5: Figure S5C,G). When expressed as a percentage of the total, lean mass of *Grb10*^*m/+*^ mice was significantly increased compared to *Dlk1*^*+/p*^ mice, both in males (Figure 7E,G) and females (see Additional file 5: Figure S5E,G). No significant differences were observed in absolute values for adipose tissue content of males (Figure 7D,G) or females (see Additional file 5: Figure S5D,G); however, when expressed as a percentage of total body mass, adipose tissue content was significantly increased in *Dlk1*^*+/p*^ compared to *Grb10*^*m/+*^ for both males (Figure 7E,G) and females (see Additional file 5: Figure S5F,G). In support of this observation, serum triglyceride concentration measured in adult males was found to be significantly elevated in *Dlk1*^*+/p*^ mice when compared to *Grb10*^*m/+*^ mice (Figure 7H,I) and the wet weight of both gonadal and renal adipose depots was significantly increased in females at three to six months of age (Table 1). Changes in lean:adipose body proportions were not accompanied by any obvious changes in white adipose tissue histomorphometry (see Additional file 6: Figure S6) or food consumption (see Additional file 7: Figure S7). *Dlk1* knockout mice derived independently of those used in this study were shown to exhibit transient adipocyte hypotrophy [53], with mean adipocyte size being reduced in the analysed adipose depots at six weeks of age, but not at sixteen weeks. Our adipose measurements were carried out on a cohort of three-month-old animals and transient adipocyte hypotrophy in younger *Dlk1*^*+/p*^ animals has not been excluded.

### Both *Grb10*^*m/+*^ and *Grb10*^*m/+*^*/Dlk1*^*+/p*^ mice exhibit enhanced glucose clearance compared to *Dlk1*^*+/p*^ mice

**Figure 8:**
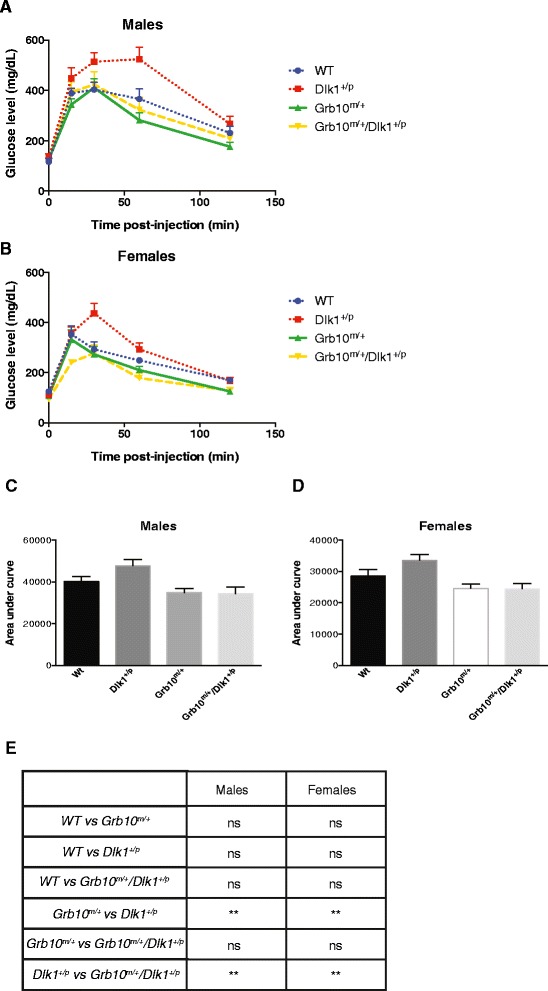
**Glucose tolerance in male and female adult mice.** Mice at three- to nine-months old were examined for their ability to clear a glucose load. Glucose clearance over time is presented graphically for males **(A)** and females **(B)**. Analysis of the area under each of the glucose concentration curves revealed that *Grb10* ^*m/+*^ and *Grb10* ^*m/+*^ */Dlk1* ^*+/p*^ mice of both males **(C)** and females **(D)** cleared glucose significantly faster than *Dlk1* ^*+/p*^ animals. **E)** Table summarising results of statistical analysis. All values represent means ± SEM and have been subject to one way ANOVA with *post hoc* Tukey’s analysis. Males: WT n = 14, *Dlk1* ^*+/p*^ n = 12, *Grb10* ^*m/+*^ n = 12 and *Grb10* ^*m/+*^ */Dlk1* ^*+/p*^ n = 13; females: WT n = 13, *Dlk1* ^*+/p*^ n = 12, *Grb10* ^*m/+*^ n = 12 and *Grb10* ^*m/+*^ */Dlk1* ^*+/p*^ n = 12; ** *P* <0.01. ANOVA, analysis of variance; SEM, standard error of the mean; WT, wild type.

Previous studies have shown that in *Grb10*^*m/+*^ knockout mice increased lean:adipose body proportions and elevated insulin signalling were associated with improved glucose tolerance and insulin sensitivity [39,42,43]. Conversely, transgenic mice overexpressing *Grb10* exhibited impaired glucose tolerance and insulin resistance [56,57]. There are no published studies of glucose or insulin tolerance in *Dlk1* knockout mice, but transgenic mice overexpressing *Dlk1* exhibit reduced adipose deposition, glucose intolerance and insulin resistance [50,51]. Consequently, we performed glucose tolerance tests (GTTs) on mice of all four genotypes used in this study (Figure 8). In all cases, following intraperitoneal injection of fasted animals with a bolus of glucose, blood glucose concentration showed an initial sharp rise followed by a gradual decline, consistent with insulin mediated glucose uptake. Compared to wild type controls, curves plotted for *Dlk1*^*+/p*^ mice showed impaired glucose clearance, while those for *Grb10*^*m/+*^ and *Grb10*^*m/+*^*/Dlk1*^*+/p*^ mice showed enhanced glucose clearance, both for males (Figure 8A) and females (Figure 8B). Comparisons of the area under each curve revealed significant differences in glucose clearance between *Dlk1*^*+/p*^ and both *Grb10*^*m/+*^ and *Grb10*^*m/+*^*/Dlk1*^*+/p*^ mice, again for both males and females (Figure 8C,D, E).

### Neonatal *Grb10*^*m/+*^ and *Grb10*^*m/+*^*/Dlk1*^*+/p*^ mice have thickened lung epithelia compared with wild type and *Dlk1*^*+/p*^ mice, whereas no differences were detected in histology of kidney and pancreas, or in skeletal morphology

**Figure 9:**
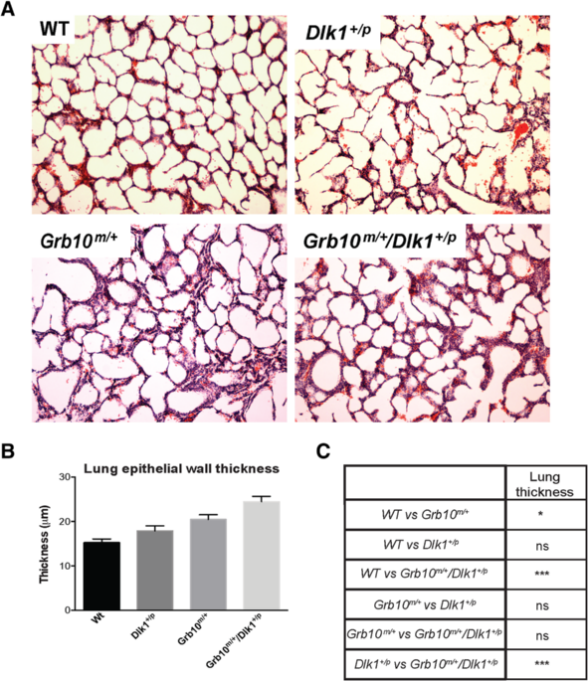
**Histological and morphometric analyses of neonatal lungs. A)** Thickened epithelial walls were observed in *Grb10* ^*m/+*^ and *Grb10* ^*m/+*^ */Dlk1* ^*+/p*^ lungs, while *Dlk1* ^*+/p*^ lungs displayed normal histology when compared to wild type littermates. The presented images are representative sections for each of the analysed genotypes (100x magnification). **B)** These observations were confirmed by morphometric analysis of epithelial wall thickness using images captured at 200x magnification. **C)** Table summarising results of statistical analysis of morphometric data. All values represent means ± SEM, analysed using one way ANOVA with Tukey’s *post-hoc* analysis. WT n = 5, *Dlk1* ^*+/p*^ n = 5, *Grb10* ^*m/+*^ n = 5 and *Grb10* ^*m/+*^ */Dlk1* ^*+/p*^ n = 5; * *P* <0.05; *** *P* <0.001. ANOVA, analysis of variance; SEM, standard error of the mean; WT, wild type.

Following the previous reports of neonatal death in a minority of *Grb10*^*m/+*^ mice that was associated with blood filled lung alveoli [38] and pulmonary defects noted in *Dlk1* knockout mice [49], we performed histological analyses of neonatal lungs (Figure 9A). Measurements of lung epithelial walls showed that both *Grb10*^*m/+*^ and *Grb10*^*m/+*^*/Dlk1*^*+/p*^ mice had significantly thicker epithelial walls when compared to either *Dlk1*^*+/p*^ or wild type animals (Figure 9B,C). Similar morphometric analysis of adult lung showed no differences between any of the four genotypes, indicating that the thickened lung epithelial wall phenotype did not persist into adulthood (data not shown).

Skeletal malformations were previously reported in *Dlk1* knockout mice [49] and the soluble form of Dlk1, fetal antigen-1 (FA1), has been identified as a novel endocrine factor regulating chondrocyte differentiation *in vitro* [58], as well as bone and adipose mass *in vivo* [59,60]. Our *Grb10* knockout mice incorporate a β-galactosidase reporter gene within the *Grb10* locus that we have previously found to recapitulate endogenous *Grb10* expression patterns in both fetal and adult tissues [17,38–40], although expression can be low at specific sites where *Grb10* appears to be regulated by STAT5 [41]. Here we employed *LacZ* staining to examine *Grb10* expression in neonatal bones, with expression readily detected in growth plates of *Grb10*^*m/+*^ and *Grb10*^*m/+*^*/Dlk1*^*+/p*^ animals, indicating a possible role for *Grb10* in growth of long bones during skeletogenesis (see Additional file 8: Figure S8A). However, staining of skeletal preparations of mice of all four genotypes with Alcian Blue and Alizarin Red to highlight cartilage and bone, respectively, revealed no obvious skeletal defects (see Additional file 8: Figure S8B), including those previously described for *Dlk1* knockout mice. This difference in the mice used by ourselves and Moon *et al.* [49] could be due to the precise nature of the engineered mutations or their maintenance on different strain backgrounds. Nevertheless, the observed expression of *Grb10* in the growth plate of long bones is consistent with a role in skeletogenesis, which could contribute to the growth phenotype of *Grb10*^*m/+*^ and *Grb10*^*m/+*^*/Dlk1*^*+/p*^ mice.

Use of the *LacZ* insert at the *Grb10* locus in our knockout mice revealed that the maternal *Grb10* allele is expressed in adult kidney, most obviously in the proximal tubules (see Additional file 9: Figure S9). This, together with the observation of altered kidney proportions in *Grb10*^*m/+*^ and *Grb10*^*m/+*^*/Dlk1*^*+/p*^ mice (Figure 4 and Table 1), prompted us to examine adult kidneys in more detail. However, we detected no obvious differences in histology of adult kidney among animals of the four genotypes used in this study (see Additional file 9: Figure S9) and in all cases there was no evidence of compromised kidney function as judged by analysis of protein content of urine samples collected from mice at three months of age (data not shown). The observed expression pattern may be relevant to the tumour suppressor role proposed for Grb10 in small cell renal cell carcinoma [61], a prevalent adult kidney cancer thought to arise predominantly from the proximal tubule [62].

## Discussion

**Figure 10:**
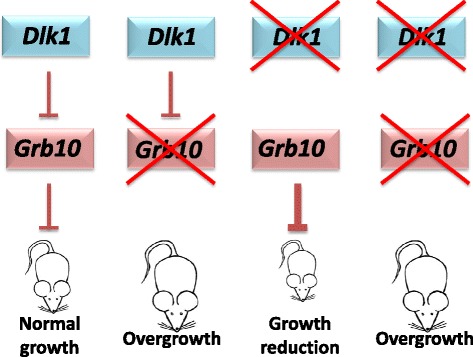
**Model of the genetic interaction between** ***Dlk1*** **and** ***Grb10*** **.** Normal growth is observed only in wild type animals, with intact *Grb10* and *Dlk1* genes. Gene knockout (indicated by an ‘X’ over the relevant genotypes) of maternal *Grb1O* (*Grb10* ^*m/+*^mice) results in overgrowth, whereas knockout of paternal *Dlk1* (*Dlk1* ^*+/p*^ mice) results in growth restriction. Simultaneous knockout of both genes (*Grb10* ^*m/+*^ */Dlk1* ^*+/p*^ mice) results in overgrowth, with associated changes in body proportions, histology and physiology, essentially as in *Grb10* ^*m/+*^ mice. Thus, *Grb10* is an inhibitor of fetal growth while *Dlk1* acts to promote growth by inhibition of *Grb10*.

Paternally-expressed *Igf2* and maternally-expressed *Igf2r* were the first imprinted genes to be identified and their opposite growth regulatory roles were quickly recognised to fit with predictions of the parental conflict hypothesis [18,19]. Genetic crosses between females heterozygous for an *Igf2r* knockout allele and males heterozygous for an *Igf2* knockout allele revealed that the growth inhibitory function of *Igf2r* could be attributed entirely to an antagonistic interaction with *Igf2* [22,23]. This conclusion derives from the observation that the growth of *Igf2r*^*m/+*^*/Igf2*^*+/p*^ double knockout offspring was indistinguishable from that of *Igf2*^*+/p*^ knockout littermates. Although not a prerequisite of the hypothesis, the fact that the opposing functions of the two genes were enacted through direct interaction of their gene products made the case for a parental ‘tug-of-war’ even more compelling [63,64]. Here, we have carried out a test for epistasis between *Grb10* and *Dlk1*, oppositely imprinted genes with contrasting effects on fetal growth, adiposity and glucose homeostasis [38,39,42,49–51,59]. We carried out detailed analyses of wild type, *Grb10*^*m/+*^ and *Dlk1*^*+/p*^ single knockout, and *Grb10*^*m/+*^*/Dlk1*^*+/p*^ double knockout mice for features related to growth and metabolic phenotypes previously established as characteristic of either single knockout. Quantitative phenotyping consistently revealed that *Grb10*^*m/+*^*/Dlk1*^*+/p*^ double knockout and *Grb10*^*m/+*^ mice closely resemble each other, such that they are never significantly different, and are distinct from wild type and *Dlk1*^*+/p*^ mice. Distinguishing phenotypic characters include growth during early life, cell cycle profiles, tissue proportions, glucose regulated metabolism and specific histological features, notably lipid storage in the liver. These findings support the notion that *Grb10* and *Dlk1* act in the same genetic pathway, with *Dlk1* acting upstream of *Grb10*. In order to promote growth *Dlk1* must inhibit *Grb10*, which is in turn a growth inhibitor (Figure 10).

Disruption of the maternal *Grb10* allele has previously been shown to result in fetal and placental overgrowth [17,38–40]. *Grb10*^*m/+*^ mice are at birth around 25% to 40% larger than wild type littermates, with significant differences in weight first apparent in the fetus at E12.5 and in the placenta at E14.5. In addition, the enlarged *Grb10*^*m/+*^ placenta was found to be significantly more efficient than the wild type placenta from E17.5 [40]. In the present study, the overgrowth phenotype of *Grb10*^*m/+*^ mice was similar in both magnitude and timing to that previously observed, with significantly increased placental efficiency again manifest from E17.5. At the three gestational stages examined *Dlk1*^*+/p*^ fetal and placental weights were consistently lower than those of wild type littermates, but not statistically significantly so. Similar data were reported recently for a different Dlk1 knockout strain [53]; however, the small decreases in fetal weight are likely to have biological significance as our *Dlk1*^*+/p*^ mice were at birth significantly smaller (approximately 20% by weight) than their wild type littermates, again as previously reported [49]. With the exception of E12.5 placentae, weights of *Dlk1*^*+/p*^ offspring were always significantly lower than those of both *Grb10*^*m/+*^ and *Grb10*^*m/+*^*/Dlk1*^*+/p*^ offspring. Importantly, *Grb10*^*m/+*^ and *Grb10*^*m/+*^*/Dlk1*^*+/p*^ offspring closely resembled each other and were never significantly different.

The close resemblance of *Grb10*^*m/+*^ and *Grb10*^*m/+*^*/Dlk1*^*+/p*^ offspring was also apparent in comparisons of tissue proportions. We have previously noted disproportionate liver overgrowth in *Grb10*^*m/+*^ pups at birth [17,38] and here this was seen in both *Grb10*^*m/+*^ and *Grb10*^*m/+*^*/Dlk1*^*+/p*^ offspring at birth and at E17.5. At these stages liver was proportionate to body size in *Dlk1*^*+/p*^ mice, whereas kidney was significantly enlarged compared to wild type controls. In both *Grb10*^*m/+*^ and *Grb10*^*m/+*^*/Dlk1*^*+/p*^ the kidneys of neonates were significantly reduced as a proportion of body weight. Brain weight remained essentially unchanged in neonates of all four genotypes, such that it was enlarged in proportion to body weight in *Dlk1*^*+/p*^ and proportionately small in *Grb10*^*m/+*^ neonates. We have noted this effect before in *Grb10*^*m/+*^ neonates, which correlates with the lack of expression from the maternal *Grb10* allele in the developing brain [17,38]. This is consistent with the *Grb10*/*Dlk1* growth regulatory pathway having a brain ‘sparing’ role [65], under conditions in which fetal growth is restricted, notably when nutrients are limiting. The underlying mechanisms are poorly understood but in *Drosophila* sparing of the CNS under starvation conditions has been linked with tissue-specific modifications in PI3K/TOR signalling [66]. The TOR (mTOR in mammals) pathway is highly conserved, with key roles in nutrient sensing and growth factor signalling [67,68]. Thus, a brain sparing role for Grb10 through the established interaction with mTOR [46,47] is plausible.

Growth rates of PMEFs derived from E14.5 fetuses showed differences between genotypes that were in keeping with the observed fetal growth phenotypes. Thus, while *Dlk1*^*+/p*^ fibroblasts proliferated slowly (though not significantly so) compared to wild type, both *Grb10*^*m/+*^ and *Grb10*^*m/+*^*/Dlk1*^*+/p*^ fibroblasts exhibited a significantly higher proliferation rate. FACS analyses of E14.5 fetus-derived fibroblasts revealed for both *Grb10*^*m/+*^ and *Grb10*^*m/+*^*/Dlk1*^*+/p*^ cells a significant reduction in the number of cells in S-phase of the cell cycle, with a concomitant increased number in G2. The same FACS profile was observed for cells derived from disaggregated E11.5 fetuses. These FACS data, together with the fibroblast proliferation rates, indicate that the enhanced growth of *Grb10*^*m/+*^ and *Grb10*^*m/+*^*/Dlk1*^*+/p*^ fetuses is associated with more rapid progress through S-phase. *Grb10* overexpression in an *in vitro* leukaemia model was associated with increased cell numbers in S-phase, increased proliferation and decreased apoptosis [69], suggesting that its role in cell cycle regulation is highly context dependent. The FACS data from E11.5 fetal cells indicates that the cell cycle changes we observed are physiologically relevant and, logically, manifest before any significant effect on fetal weight can be detected (at E12.5). This is analogous to the early detection of cell cycle changes in *Igf2* knockout mice (*Igf2*^*+/p*^) [7], but whereas the major cell cycle changes in *Igf2*^*+/p*^ fetuses were focused within a narrow developmental window (E9-E10), our PMEF data suggest that altered cell cycle regulation in *Grb10*^*m/+*^ and *Grb10*^*m/+*^*/Dlk1*^*+/p*^ fetuses extends at least until E14.5.

Body and tissue weights were analysed at three further post-natal stages: one week, three to six months and more than six to nine months. Weights and tissue proportions were consistently similar between *Grb10*^*m/+*^ and *Grb10*^*m/+*^*/Dlk1*^*+/p*^ animals and were unlike those of wild type and/or *Dlk1*^*+/p*^ animals. Notably, adult *Dlk1*^*+/p*^ mice exhibited a tendency to accumulate excess adipose that is in keeping with a previous report [49], although in that case increased adiposity was revealed after placing the mice on a high fat diet. A third *Dlk1* knockout strain, maintained on a regular diet and consistently having a reduced body weight (measured between 7 and 42 weeks of age) in comparison with wild type controls, exhibited no significant changes in the weights of several adipose depots [53]. In contrast, *Grb10*^*m/+*^ and also *Grb10*^*m/+*^*/Dlk1*^*+/p*^ adults tended to have greater lean mass, although this was not as pronounced as in previous studies of *Grb10*^*m/+*^ animals [39,42–44], which we suspect can be attributed to the genotype of the *Grb10* heterozygote mothers used in different crosses. Here, the females used as mothers were themselves *Grb10*^*m/+*^ animals and, therefore, large at birth and lean as adults, whereas the *Grb10*^*+/p*^ mothers used in previous studies were effectively wild type in growth and physiology. This needs to be verified but is interesting because it indicates that the environment provided to offspring by *Grb10*^*m/+*^ and *Grb10*^*+/p*^ mothers during pregnancy and/or the post-natal suckling period is different and has a long-standing effect on offspring physiology. This is consistent with a role for *Grb10* in developmental programming of offspring growth and metabolism, as previously suggested by ourselves [41] and others [70,71].

The more subtle change in lean:adipose proportions seen in *Grb10*^*m/+*^ and *Grb10*^*m/+*^*/Dlk1*^*+/p*^ animals in this study is also noteworthy when considering that animals of both genotypes exhibited an enhanced ability to clear a glucose load similar in magnitude to that previously reported in Grb10^*m/+*^ mice [39,43]. Previous studies have shown that *Grb10*^*m/+*^ mice exhibit elevated insulin signalling in muscle and white adipose tissue (WAT), but it was unclear whether this made a major contribution to their improved glucose tolerance because they also had significantly increased lean/muscle mass [39,42–44]. Here, the clear improvement in glucose tolerance with a less dramatic increase in muscle mass suggests that enhanced insulin signalling does have a major effect on glucose homeostasis in *Grb10*^*m/+*^ (and *Grb10*^*m/+*^*/Dlk1*^*+/p*^) animals. This conclusion is supported by a recent report showing that cultured primary myotubes derived from Grb10^−/−^ mice exhibit enhanced glucose uptake and insulin signalling [54].

The changes in adiposity in *Dlk1*^*+/p*^ (increased), *Grb10*^*m/+*^ and *Grb10*^*m/+*^*/Dlk1*^*+/p*^ (both decreased) animals were reflected in serum triglyceride levels and in histological changes seen in liver at different developmental stages. *Grb10*^*m/+*^ and *Grb10*^*m/+*^*/Dlk1*^*+/p*^ animals had at birth disproportionately enlarged livers with a high lipid content compared to wild type and *Dlk1*^*+/p*^ livers. At one week of age, evidence of high lipid content could be seen in only around 50% of *Grb10*^*m/+*^ and *Grb10*^*m/+*^*/Dlk1*^*+/p*^ livers, consistent with the down-regulation of *Grb10* in liver and other tissues post-natally [39]. In adulthood, *Grb10*^*m/+*^ and *Grb10*^*m/+*^*/Dlk1*^*+/p*^ livers had a low lipid content and weighed less than those of wild type controls, whereas *Dlk1*^*+/p*^ livers had a high lipid content. Dlk1 has established roles in influencing proliferation and differentiation of several tissues, including adipose [50,72–74] and skeletal muscle [55]. The developmental mechanisms through which Grb10 regulates growth and tissue proportions are less well understood, although recent evidence indicates that primary cultures of muscle cells from Grb10^−/−^ mice exhibit increased rates of proliferation and differentiation [54]. Our data indicate roles for both Grb10 and Dlk1 in regulating adipose distribution that is consistent with the recognised role for Dlk1 in inhibiting adipocyte differentiation. It will be important in the future to focus greater attention on Grb10 as a potential regulator of the balance between cell proliferation and differentiation, not least in the tissues where it has an established physiological role, including adipose [39,42,48], skeletal muscle [39,42,44] and now liver.

The molecular basis of the interaction between Grb10 and Dlk1 is currently unknown and may not be direct as in the case of Igf2 and Igf2r. Since our genetic experiments indicate that *Dlk1* operates upstream of *Grb10* (Figure 10), the most parsimonious prediction would be that Dlk1 acts as a ligand for a cell surface receptor that in turn interacts with Grb10. The Grb10 adaptor protein is capable of interacting with many receptors and downstream signalling components, including mTOR [45–48]. Conversely, the receptor(s) for Dlk1 are unknown. Although Dlk1 shares amino acid sequence homology with members of the Delta/Serrate family of ligands it lacks the motifs known to be important for interaction with the Notch family of receptors [52]. Despite this there is *in vitro* evidence that Dlk1 may act as an inhibitor of the NOTCH1 receptor, but also that it can influence other signalling pathways, including ERK/MAPK, AKT/PI3K and mTOR [58,75–78] signalling. Thus, there is ample scope for Dlk1 and Grb10 to interact in key growth factor signalling pathways. Further work on our single and double knockout mouse models will be required to establish the molecular pathway between Dlk1 and Grb10.

## Conclusions

Based on the accumulated evidence we propose that *Grb10* and *Dlk1* may define a mammalian growth-regulatory axis that is separate from the IGF pathway, yet is similar in having as key components a pair of oppositely imprinted genes with antagonistic functions. This is only the second clear example of a pair of imprinted genes with such antagonistic roles, after *Igf2* and *Igf2r*.

## Methods

### Mice

The *Grb10Δ2-4* knockout mouse strain (herein referred to as *Grb10*^*m/+*^) was generated as described in [38] and stocks maintained on a mixed C57BL/6 x CBA genetic background. *Dlk1Δ1-3*^*+/p*^ knockout mice (herein *Dlk1*^*+/p*^) were generated as described in Raghunandan *et al*. [79] and stock maintained on a C57BL/6 genetic background. Both strains were housed as previously described [80]. In order to generate experimental animals, *Grb10*^*m/+*^females were crossed with *Dlk1*^*+/p*^ male mice, thereby taking advantage of the imprinted status of each gene to generate in equal proportions mice of four genotypes; wild type (WT or *Grb10*^*+/+*^*; Dlk1*^*+/+*^*)*, *Grb10*^*m/+*^and *Dlk1*^*+/p*^ single knockouts, and *Grb10*^*m/+*^*/Dlk1*^*+/p*^ double knockouts. Siblings of each genotype were used in all subsequent analyses, thereby controlling for age and genetic background, with data from males and females analysed separately for most comparisons. Placentae and fetuses were collected and weighed at 12, 14 and 17 days after the observation of a cervical plug, designated embryonic days E12.5, E14.5 and E17.5, respectively. Animals were genotyped by PCR using primers described previously for both *Grb10* [38] and *Dlk1* [79] alleles. All experiments were carried out under license from the United Kingdom Home Office.

### Tissue dissection and histological procedures

Mice were humanely culled and their total body weights recorded, as well as organ weights following dissection. Organs destined for wax embedding were fixed in 4% (w/v) paraformaldehyde (PFA) in PBS at 4°C overnight, processed using a Leica TP 1020 machine, and sections cut at approximately 6 μm using a microtome (Leica RM 2155) prior to staining with haematoxylin and eosin (H & E) or periodic acid – Schiff stain, as previously described [81]. Organs destined for cryosectioning were fixed for 30 minutes in 2% PFA, 0.2% glutaraldehyde in PBS, transferred into ice cold 30% (w/v) sucrose and incubated at 4°C overnight, then embedded in optimal cutting temperature (OCT) compound (VWR International, Lutterworth, UK) and sections cut at approximately 10 μm using a cryostat (CM1850; Leica). For lipid staining a working solution of Oil Red O (Fisher Scientific, Loughborough, UK) in 60% isopropanol was heated and stirred at 100°C for five minutes and then filtered using 25 μm Whatman filter paper (Camlab, Cambridge, UK), first when hot and then again when cool. Cryosections were air-dried and rinsed with 60% isopropanol prior to incubating in Oil Red O solution for 15 minutes. Sections were then rinsed with 60% isopropanol and gently washed under running tap water until clear. Slides were counterstained with Mayer’s haematoxylin or Light Green (Lamb) for 30 seconds, washed under running tap water for approximately three minutes and mounted in gel mounting medium (VectaShield, Vector Laboratories, Peterborough, UK). ImageJ software was used to calculate the liver surface stained by Oil Red O. First, the colour channels in the picture were split and the green channel was chosen for analysis. Next, the threshold was adjusted so the highlighted area most accurately resembled the red staining in the original picture and the highlighted area was calculated. Cryosections for β-galactosidase gene expression analysis were fixed for 30 minutes in 4% PFA and then incubated in 1 mg/ml 5-bromo-4-chloro-3-indolyl-beta-D-galactopyranoside (X-gal) in stain base (5 mM K_4_Fe(CN)_6_, 5 mM K_3_Fe(CN)_6_.3H_2_O, 2 mM MgCl_2_, 0.01% (w/v) sodium deoxycholate, 0.02% (v/v) Igepal CA-630 in PBS) overnight at 37°C. Following incubation, sections were rinsed in PBS and stained with Nuclear Fast Red (Vector Laboratories). Stained sections were viewed under a Nikon Eclipse E800 compound microscope and pictures taken using a Nikon digital Sight DS-U1 camera operated with NIS Elements D 2.30 software.

### Morphometric analysis

Pictures of lung, kidney and adipose tissue sections from three-month-old male mice were captured at 200x magnification and overlaid with an 8 x 4 grid. The whole section was divided into 32 squares of equal size and the same five specific squares from each slide were used for morphometric measurements or cell counting.

### Alcian blue/Alizarin red staining

For skeletal analysis, we adapted the method of McLeod [82]. Skin and dense organs were removed from carcasses, fixed in 100% ethanol for three days and then in acetone for one day. Skeletons were washed in water purified using a Milli-Q Biocel (Millipore, Billerica, MA, USA) for several minutes before staining in colour solution (1 part 0.3% Alcian Blue in 70% ethanol: 1 part 0.1% Alizarin Red in 95% ethanol: 1 part glacial acetic acid: 17 parts 70% ethanol) for three days at room temperature. Skeletons were then submerged in purified water for several minutes and soft tissues were cleared in 1% KOH until the skeleton was visible. They were then transferred serially into 50% and 80% glycerine for approximately 24 hours each, and finally transferred into 100% glycerine.

### Dual energy X-ray absorptiometry and measurement of blood serum triglyceride levels

Carcasses from mice between three to nine months of age were analysed by DXA using a PIXImus scanner (Lunar, Madison, WI, USA) with small-animal software, essentially as previously described [39]. Readouts were recorded of total body area, lean and adipose tissue weight, bone mineral density and bone mineral content. Lean and adipose tissue weights were expressed as percentages of total body weight before performing statistical analysis. Triglyceride levels were measured in three-month-old male mice fasted for 16 hours prior to the start of the experiment. Between 50 μl and 100 μl of blood was obtained from the tail tip. Triglyceride levels were measured using a Triglyceride Assay Kit (Cayman Chemical Company, Ann Arbor, MI, USA) according to manufacturer’s instructions.

### Food consumption, glucose tolerance tests and proteinurea assays

Food consumption measurements and also glucose tolerance tests performed to assess whole body glucose clearance were carried out as previously described [39] on mice between three to six and more than six to nine months of age. Food consumption was calculated in direct proportion to body mass, and also using the ratio of 2/3x body mass as a scaling component to take account of basal metabolic rate which varies as a function of body mass [83]. Assays of urine for the appearance of elevated protein levels as a measure of impaired kidney function were performed using SDS-PAGE, essentially as described previously [84] on samples from male mice at three months of age.

### Primary mouse embryonic fibroblast analyses

PMEF cells were derived from E14.5 embryos and maintained according to methods described in [85]. Cell number per 1 ml was determined using a haemocytometer. Following the third passage, 1 x 10^4^ cells were seeded per well of a standard 24-well plate and an initial count of the attached cells was taken 24 hours later. After this point cells from four wells were counted every 48 hours over an 11 day period in three separate experiments and the results used to calculate mean values. Cell growth was calculated as a percentage of the initial seeding density.

### Propidium iodide staining of whole embryo cell extracts and primary mouse embryonic fibroblasts for fluorescence activated cell sorting analysis

Analysis of cells from disaggregated embryos was carried out essentially as described in [7]. Embryonic day 11.5 (E11.5) embryos were dissected from the uterus and washed several times with filtered, ice cold PBS. Embryos were disaggregated using a pestle inside a 1.5 ml tube, gently resuspended and centrifuged for 10 minutes at 500 *g*. Cell resuspension in ice cold PBS was repeated twice, with an intervening centrifugation, and then cells were gently dissociated by filtering through a 40 μm nylon cell strainer (BD Biosciences, San Jose, CA, USA). PMEFs reaching approximately 70% confluency were washed with PBS, trypsinised by adding 1 ml of 0.25% trypsin-ethylenediaminetetraacetic acid (Fisher Scientific, Loughborough, UK) solution per 25 cm^2^ and washed twice in ice cold PBS.

Embryonic cell suspensions and PMEFs were stained for 30 minutes at 37°C in propidium iodide solution (50 μg/ml propidium iodide, 100 μg/ml RNAse A, 0.1% Igepal CA-630 and 50 μg/ml trisodium citrate in PBS). Stained cells were then washed in PBS and 100,000 fluorescence events were collected using a FACS Canto instrument (BD Biosciences) at the excitation wavelength of 488 nm and an emission wavelength of 600 nm. Cells were analysed for cell size (forward scatter versus side scatter) and cell cycle position (propidium iodide concentration). Data were analysed using FlowJo software (v7.6, FlowJo Software, Ashland, OR, USA).

### Statistical analysis

GraphPad Prism (v5, GraphPad Software, La Jolla, CA, USA) was used for statistical analysis. Results were subject to one-way analysis of variance (ANOVA), and a *post-hoc* Tukey test was applied. Graphs represent arithmetic means ± standard error of the mean (SEM). Differences with *P* values of <0.05 were considered to be statistically significant. For the purpose of clarity, no stars representing significant differences are depicted in any of the graphs but all possible statistical comparisons and any significant differences are shown in tables that accompany each graph, indicated as: * *P* <0.05; ** *P* <0.01; *** *P* <0.001.

## Additional files

Additional file 1: Figure S1. Analyses of fetal and placental wet weights at E12.5 and E17.5A). A) At E12.5 both *Grb10* ^*m/+*^ and *Grb10* ^*m/+*^ */Dlk1* ^*+/p*^ fetuses, but not placentae, were significantly heavier than wild type and *Dlk1* ^*+/p*^ littermates. B) At E17.5 *Grb10* ^*m/+*^ and *Grb10* ^*m/+*^ */Dlk1* ^*+/p*^ fetuses were significantly heavier than wild type and *Dlk1* ^*+/p*^ littermates, and in addition *Grb10* ^*m/+*^ and *Grb10* ^*m/+*^ */Dlk1* ^*+/p*^ placentae were heavier than *Dlk1* ^*+/p*^ placentae. C) Table summarising results of statistical analysis in A and B. All values represent means ± SEM, one way ANOVA with Tukey’s *post-hoc* analysis. For E12.5 WT n = 23, *Dlk1* ^*+/p*^ n = 13, *Grb10* ^*m/+*^ n = 13, *Grb10* ^*m/+*^ */Dlk1* ^*+/p*^ n = 16; for E17.5 WT n = 4, *Dlk1* ^*+/p*^ n = 8, *Grb10* ^*m/+*^ n = 6, *Grb10* ^*m/+*^ */Dlk1* ^*+/p*^ n = 8; * *P* <0.05; ** *P* <0.01; *** *P* <0.001. Additional file 2: Figure S2. Cell size analysis by FACS of E14.5 PMEFs and disaggregated E11.5 fetuses. A) Cultured E14.5-derived PMEFs, at passage 3, were stained with propidium iodide and cell size determined by FACS for 100,000 cells per sample. Cells were allocated to one of four arbitrary gates (0 to 25 k, >25 to 50 k, >50 to 75 k, >75 to 100 k) to allow statistical comparison of cell size distribution. B) Table summarising results of statistical analysis of data in A. All values represent means ± SEM, one way ANOVA with Tukey’s *post-hoc* analysis. WT n = 7, *Dlk1* ^*+/p*^ n = 7, *Grb10* ^*m/+*^ n = 7, *Grb10* ^*m/+*^ */Dlk1* ^*+/p*^ n = 6. No significant differences were observed between cells of any of the four genotypes. C) Cells derived directly from wild type, *Dlk1* ^*+/p*^, *Grb10* ^*m/+*^ and *Grb10* ^*m/+*^ */Dlk1* ^*+/p*^ E11.5 fetuses were stained with propidium iodide and cell size determined by FACS for 100,000 cells per sample. Cells were allocated to one of four arbitrary gates (0 to 25 k, >25 to 50 k, >50 to 75 k, >75 to 100 k) to allow statistical comparison of cell size distribution. D) Table summarising results of statistical analysis of data in C. All values represent means ± SEM, one way ANOVA with Tukey’s *post-hoc* analysis. WT n = 7, *Dlk1* ^*+/p*^ n = 7, *Grb10* ^*m/+*^ n = 6, *Grb10* ^*m/+*^ */Dlk1* ^*+/p*^ n = 8. No significant differences were observed between cells of any of the four genotypes. Additional file 3: Figure S3. Whole body and organ wet weight analysis of wild type, *Dlk1* ^*+/p*^ *, Grb10* ^*m/+*^ and *Grb10* ^*m/+*^ */Dlk1* ^*+/p*^ neonates. A) *Grb10* ^*m/+*^ and *Grb10* ^*m/+*^ */Dlk1* ^*+/p*^ animals showed whole body overgrowth, whereas *Dlk1* ^*+/p*^ mice were significantly growth retarded when compared to wild type and *Dlk1* ^*+/p*^ mice. Note, this graph is the same as that shown in Figure 4A, reproduced here for convenience. B) *Grb10* ^*m/+*^ and *Grb10* ^*m/+*^ */Dlk1* ^*+/p*^ mice had overgrown brains when compared to wild type and *Dlk1* ^*+/p*^ animals. C) Significant enlargement of the livers was noted in *Grb10* ^*m/+*^ and *Grb10* ^*m/+*^ */Dlk1* ^*+/p*^ mice compared to wild type and *Dlk1* ^*+/p*^ animals. D) Significant overgrowth of kidneys was seen in *Grb10* ^*m/+*^ */Dlk1* ^*+/p*^ mice compared to *Dlk1* ^*+/p*^. E) Significant enlargement of the lungs was observed in *Grb10* ^*m/+*^ and *Grb10* ^*m/+*^ */Dlk1* ^*+/p*^ mice compared to wild type and *Dlk1* ^*+/p*^ animals. F) *Grb10* ^*m/+*^ and *Grb10* ^*m/+*^ */Dlk1* ^*+/p*^ mice exhibited significantly enlarged hearts compared to wild type and *Dlk1* ^*+/p*^ animals. G) Table summarising results of statistical analysis. All values represent means ± SEM, analysed using one way ANOVA with Tukey’s *post-hoc* analysis. WT n = 19, *Dlk1* ^*+/p*^ n = 36, *Grb10* ^*m/+*^ n = 23, *Grb10* ^*m/+*^ */Dlk1* ^*+/p*^ n = 22; * p < 0.05; *** *P* <0.001. Additional file 4: Figure S4. Histology of neonatal and adult livers stained with H & E. A) Histological examination of neonatal livers stained with H & E revealed that *Dlk1* ^*+/p*^ sections were indistinguishable from wild type, but *Grb10* ^*m/+*^ and *Grb10* ^*m/+*^ */Dlk1* ^*+/p*^ livers showed the presence of abundant white, round spaces (WT n = 5, *Dlk1* ^*+/p*^ n = 6, *Grb10* ^*m/+*^ n = 4 and *Grb10* ^*m/+*^ */Dlk1* ^*+/p*^ n = 5). B) There were no differences in wild type, *Grb10* ^*m/+*^ and *Grb10* ^*m/+*^ */Dlk1* ^*+/p*^ adult livers (from males at three months of age); however, the presence of large and abundant white, round spaces was noted in *Dlk1* ^*+/p*^ livers (n = 5 for each genotype). Presented images (in A and B) show representative sections for each of the analysed genotypes. Additional file 5: Figure S5. DXA analysis of adult wild type, *Grb10* ^*m/+*^, *Dlk1* ^*+/p*^ and *Grb10* ^*m/+*^ */Dlk1* ^*+/p*^ female mice. Carcasses of female animals three- to nine-months-old were analysed by DXA. A) No differences were found in bone mineral density (BMD). B) Bone mineral content was significantly reduced in *Dlk1* ^*+/p*^ mice in comparison to wild type. C) Total lean tissue mass was significantly elevated in *Grb10* ^*m/+*^ animals when compared to *Dlk1* ^*+/p*^ D) No changes were seen in total fat tissue content. E) Lean mass as a percentage of total body mass was significantly increased in *Grb10* ^*m/+*^ mice in comparison to *Dlk1* ^*+/p*^ mice. F) Fat mass as a percentage of total body mass was significantly reduced in *Grb10* ^*m/+*^ mice when compared to *Dlk1* ^*+/p*^. G) Table summarising results of statistical analysis. All values represent means ± SEM and have been subject to one way ANOVA with *post hoc* Tukey’s analysis. WT n = 13, *Dlk1* ^*+/p*^ n = 12, *Grb10* ^*m/+*^ n = 12 and *Grb10* ^*m/+*^ */Dlk1* ^*+/p*^ n = 12; * *P* <0.05; ** *P* <0.01. Additional file 6: Figure S6. Analysis of white adipose tissue sections from three-month-old mice. A) H & E stained sections of white adipose tissue. No obvious differences were noted between any of the analysed genotypes. Images are representative sections viewed at 100x magnification for each of the analysed genotypes, WT n = 6, *Dlk1* ^*+/p*^ =5, *Grb10* ^*m/+*^ n = 6 and *Grb10* ^*m/+*^ */Dlk1* ^*+/p*^ n = 6. B-E) Morphometric analysis was carried out on images captured at 200x magnification, used to analyse adipocyte cell numbers (B) and cell areas (C), as well as mean (D) and maximum cell widths (E). F) Table summarising results of statistical analysis. All values represent means ± SEM and have been subject to one way ANOVA with *post hoc* Tukey’s analysis. WT n = 5, *Dlk1* ^*+/p*^ n = 5, *Grb10* ^*m/+*^ n = 5 and *Grb10* ^*m/+*^ */Dlk1* ^*+/p*^ n = 5. No significant differences (ns; *P* >0.05) were found for any of the analysed parameters. Additional file 7: Figure S7. Analysis of food consumption. Food intake was monitored over a period of two weeks. Total consumption is shown for males (A) and females (B) and food consumption rates have been calculated as food consumed per gram of animal body weight per day to the power of 2/3 (to take account of basal metabolic rate as a function of body mass [83]), again for males (C) and females (D). E) Table summarising results of statistical analysis. All values represent means ± SEM and have been subject to one way ANOVA with *post hoc* Tukey’s analysis. Males: WT n = 14, *Dlk1* ^*+/p*^ =12, *Grb10* ^*m/+*^ n = 12 and *Grb10* ^*m/+*^ */Dlk1* ^*+/p*^ n = 13; females: WT n = 13, *Dlk1* ^*+/p*^ n = 12, *Grb10* ^*m/+*^ n = 12 and *Grb10* ^*m/+*^ */Dlk1* ^*+/p*^ n = 12. No significant differences (ns, *P* >0.05) were found in either the total amount of food consumed by males and females or in the rate of food consumption. Additional file 8: Figure S8. Skeletal analyses. A) In sections of neonatal wild type, *Grb10* ^*m/+*^, *Dlk1* ^*+/p*^ and *Grb10* ^*m/+*^ */Dlk1* ^*+/p*^ femurs *LacZ* (blue) staining was detected in *Grb10* ^*m/+*^ and *Grb10* ^*m/+*^ */Dlk1* ^*+/p*^ mice, specifically in growth plates and muscle tissue adjacent to the long bones, but not in wild type and *Dlk1* ^*+/p*^ mice, which do not carry an integrated *LacZ* reporter gene. The images show the distal growth plate (GP) of the femur, with associated muscle (M) and in some cases the knee joint and adjacent growth plate of the tibia. Presented images (400x magnification) show representative sections for each of the analysed genotypes. WT n = 4, *Dlk1* ^*+/p*^ n = 4, *Grb10* ^*m/+*^ n = 6 and *Grb10* ^*m/+*^ */Dlk1* ^*+/p*^ n = 3. B) Alcian Blue (cartilage) and Alizarin Red (bone) staining of skeletons from mice at two weeks of age revealed no obvious differences, including in the ribs and sternum previously reported to be malformed in *Dlk1* knockout mice (Moon *et al*. [49]). Additional file 9: Figure S9. Histology of adult kidney. In kidney sections from three-month-old males *LacZ* (blue) staining was observed in proximal tubules (characterised by distinctive brush border cells; (BB) of *Grb10* ^*m/+*^ and *Grb10* ^*m/+*^ */Dlk1* ^*+/p*^, but not WT or *Dlk1* ^*+/p*^, mice. Presented images show representative sections for each of the analysed genotypes, counterstained with nuclear fast red (200x magnification). WT n = 4, *Dlk1* ^*+/p*^ n = 3, *Grb10* ^*m/+*^ n = 3 and *Grb10* ^*m/+*^ */Dlk1* ^*+/p*^ n = 3.

## Abbreviations

ANOVA: analysis of variance
BMC: bone mineral content
BMD: bone mineral density
CDKN1C: cyclin-dependent kinase inhibitor 1C
CI-MPR: cation-independent mannose 6-phosphate receptor
CNS: central nervous system
Dlk1: Delta-like 1
DXA: dual energy X-ray absorptiometry
E11.5 to E17,5: mouse embryonic days 11.5 to 17.5
EDTA: ethylenediaminetetraacetic acid
FACS: fluorescence activated cell sorting
G1-phase: cell cycle growth phase 1
G2-phase: cell cycle growth phase 2
Grb10: growth factor receptor-bound protein 10
H & E: haematoxylin and eosin
IGF: insulin-like growth factor
Igf2: insulin-like growth factor 2
Igf2r: insulin-like growth factor type 2 receptor
Insr: insulin recptor
m-TOR: mammalian target of rapamycin
OCT: optimal cutting temperature
PBS: phosphate-buffered saline
PFA: paraformaldehyde
PMEF: primary mouse embryonic fibroblast
pref-1: preadipocyte factor-1
SEM: standard error of the mean
S-phase: cell cycle DNA synthesis phase
WAT: white adipose tissue
X-gal: 5-bromo-4-chloro-3-indolyl-beta-D-galactopyranoside
