# Supplementary material for: Antagonistic roles in fetal development and adult physiology for the oppositely imprinted Grb10 and Dlk1 genes
Source: BMC Biol. 2014 Dec 31;12:771. doi: 10.1186/s12915-014-0099-8 (PMC4280702; doi:10.1186/s12915-014-0099-8)
Supplement: Additional file 1: Figure S1. — Analyses of fetal and placental wet weights at E12.5 and E17.5A). A) At E12.5 both Grb10 m/+ and Grb10 m/+ /Dlk1 +/p fetuses, but not placentae, were significantly heavier than wild type and Dlk1 +/p littermates. B) At E17.5 Grb10 m/+ and Grb10 m/+ /Dlk1 +/p fetuses were significantly heavier than wild type and Dlk1 +/p littermates, and in addition Grb10 m/+ and Grb10 m/+ /Dlk1 +/p placentae were heavier than Dlk1 +/p placentae. C) Table summarising results of statistical analysis in A and B. All values represent means ± SEM, one way ANOVA with Tukey’s post-hoc analysis. For E12.5 WT n = 23, Dlk1 +/p n = 13, Grb10 m/+ n = 13, Grb10 m/+ /Dlk1 +/p n = 16; for E17.5 WT n = 4, Dlk1 +/p n = 8, Grb10 m/+ n = 6, Grb10 m/+ /Dlk1 +/p n = 8; * P <0.05; ** P <0.01; *** P <0.001. [file 12915_2014_99_MOESM1_ESM.pdf]

A

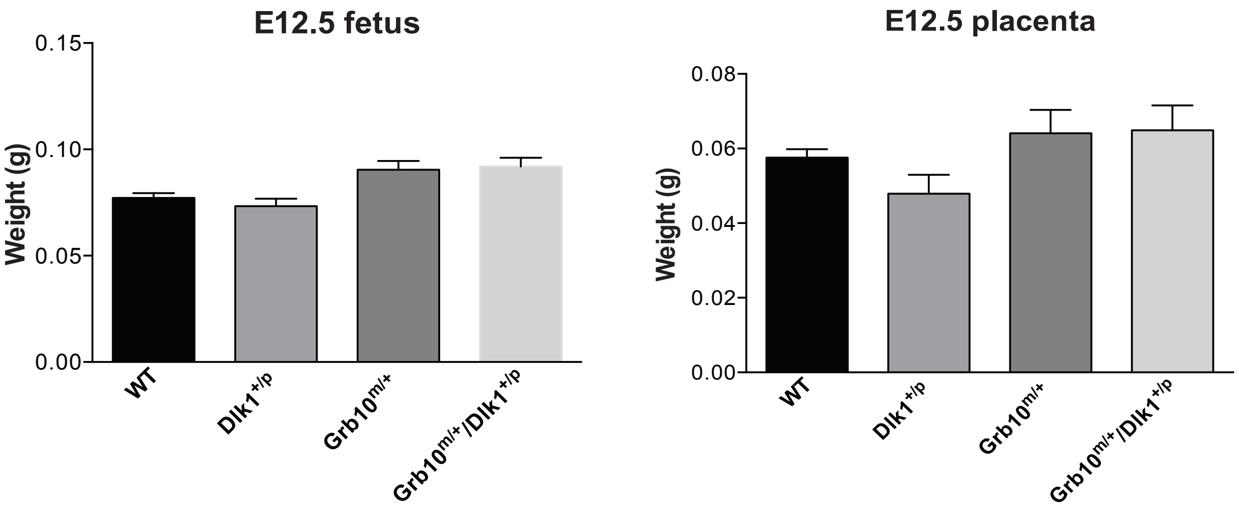

B

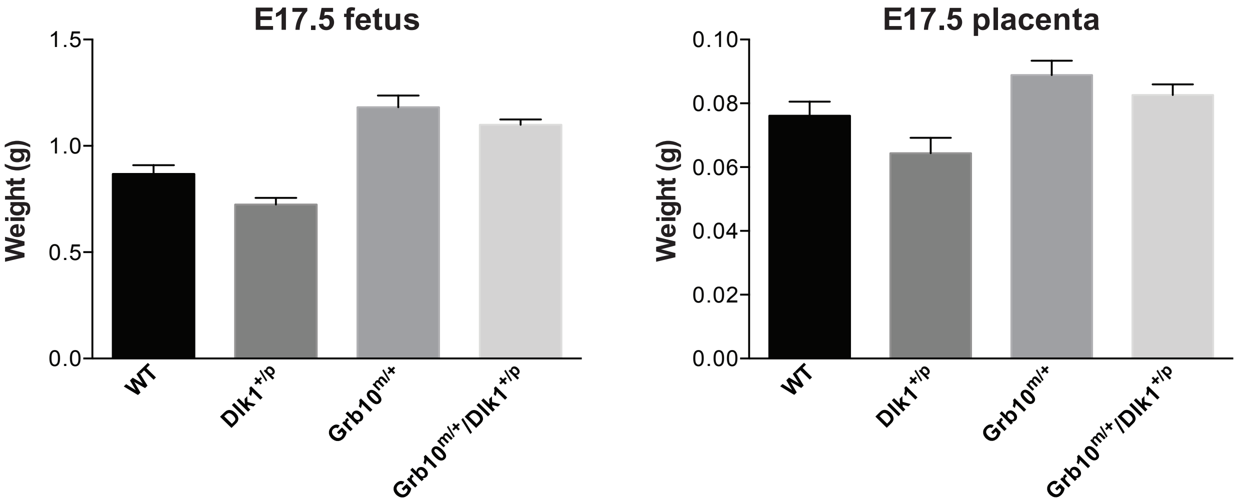

C

	E12.5 fetus	E12.5 placenta	E17.5 fetus	E17.5 placenta
WT vs $Grb10^{m/+}$	*	ns	***	ns
WT vs $Dlk1^{+/p}$	ns	ns	ns	ns
WT vs $Grb10^{m/+}/Dlk1^{+/p}$	**	ns	**	ns
$Grb10^{m/+}$ vs $Dlk1^{+/p}$	*	ns	***	**
$Grb10^{m/+}$ vs $Grb10^{m/+}/Dlk1^{+/p}$	ns	ns	ns	ns
$Dlk1^{+/p}$ vs $Grb10^{m/+}/Dlk1^{+/p}$	**	ns	***	*
