# Supplementary material for: Antagonistic roles in fetal development and adult physiology for the oppositely imprinted Grb10 and Dlk1 genes
Source: BMC Biol. 2014 Dec 31;12:771. doi: 10.1186/s12915-014-0099-8 (PMC4280702; doi:10.1186/s12915-014-0099-8)
Supplement: Additional file 2: Figure S2. — Cell size analysis by FACS of E14.5 PMEFs and disaggregated E11.5 fetuses. A) Cultured E14.5-derived PMEFs, at passage 3, were stained with propidium iodide and cell size determined by FACS for 100,000 cells per sample. Cells were allocated to one of four arbitrary gates (0 to 25 k, >25 to 50 k, >50 to 75 k, >75 to 100 k) to allow statistical comparison of cell size distribution. B) Table summarising results of statistical analysis of data in A. All values represent means ± SEM, one way ANOVA with Tukey’s post-hoc analysis. WT n = 7, Dlk1 +/p n = 7, Grb10 m/+ n = 7, Grb10 m/+ /Dlk1 +/p n = 6. No significant differences were observed between cells of any of the four genotypes. C) Cells derived directly from wild type, Dlk1 +/p, Grb10 m/+ and Grb10 m/+ /Dlk1 +/p E11.5 fetuses were stained with propidium iodide and cell size determined by FACS for 100,000 cells per sample. Cells were allocated to one of four arbitrary gates (0 to 25 k, >25 to 50 k, >50 to 75 k, >75 to 100 k) to allow statistical comparison of cell size distribution. D) Table summarising results of statistical analysis of data in C. All values represent means ± SEM, one way ANOVA with Tukey’s post-hoc analysis. WT n = 7, Dlk1 +/p n = 7, Grb10 m/+ n = 6, Grb10 m/+ /Dlk1 +/p n = 8. No significant differences were observed between cells of any of the four genotypes. [file 12915_2014_99_MOESM2_ESM.pdf]

A

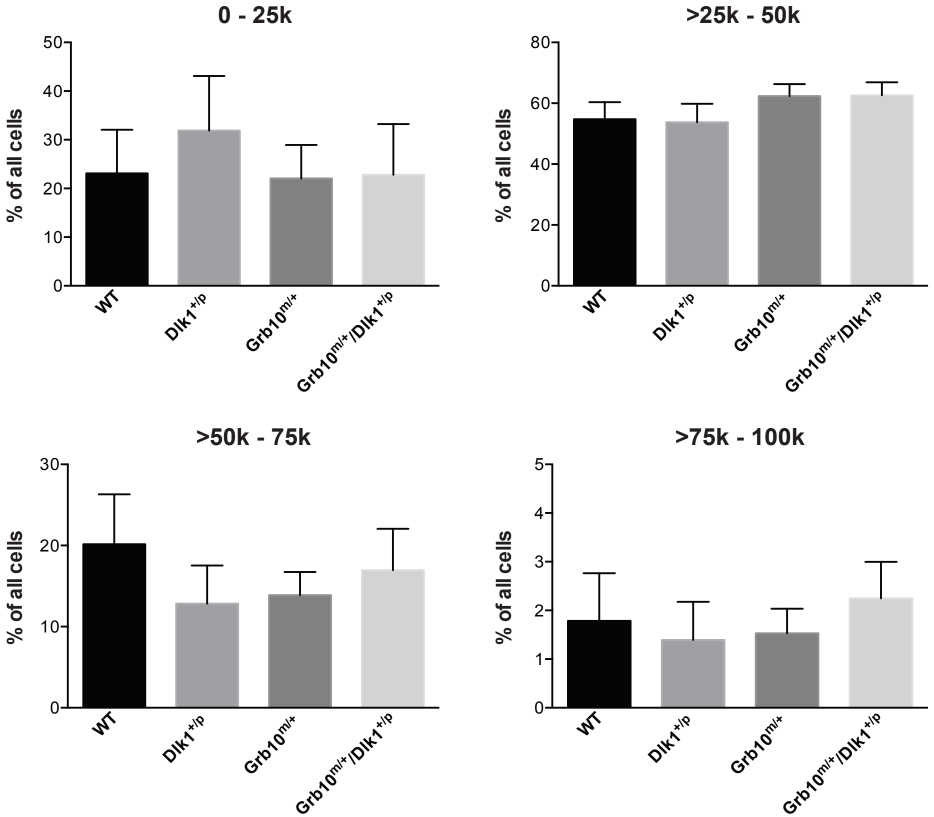

C

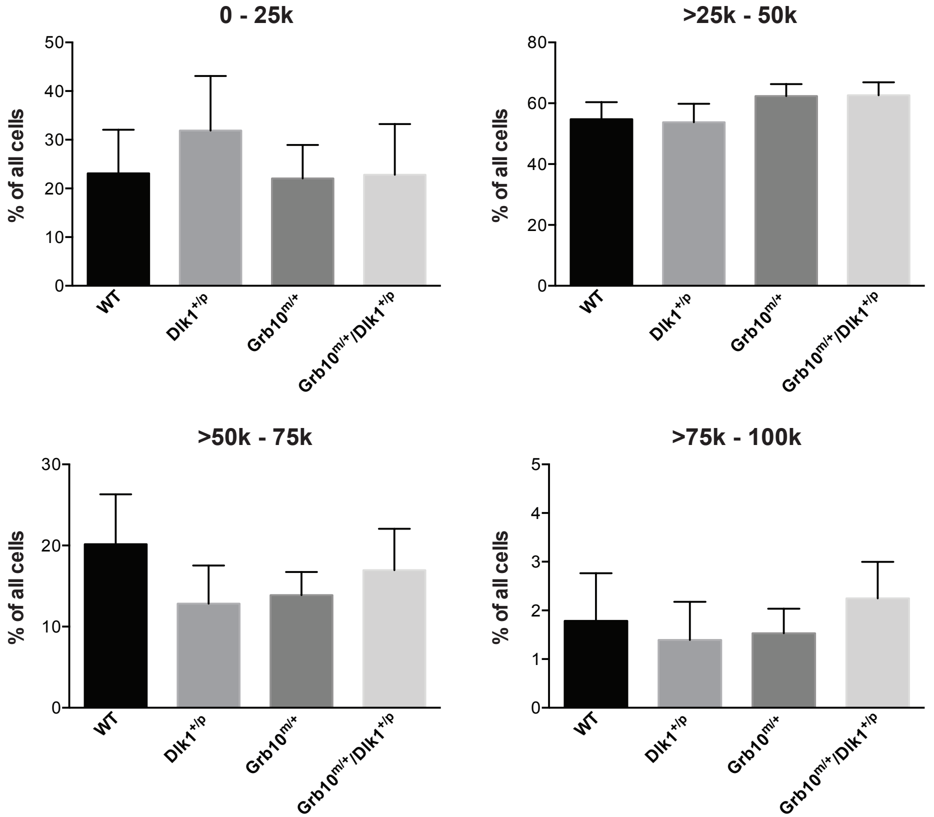

B

	0-25k	>25k-50k	>50k-75k	>75k-100k
WT vs Grb10$^{m/+}$	ns	ns	ns	ns
WT vs Dlk1$^{+/p}$	ns	ns	ns	ns
WT vs Grb10$^{m/+}$/Dlk1$^{+/p}$	ns	ns	ns	ns
Grb10$^{m/+}$ vs Dlk1$^{+/p}$	ns	ns	ns	ns
Grb10$^{m/+}$ vs Grb10$^{m/+}$/Dlk1$^{+/p}$	ns	ns	ns	ns
Dlk1$^{+/p}$ vs Grb10$^{m/+}$/Dlk1$^{+/p}$	ns	ns	ns	ns

D

	0-25k	>25k-50k	>50k-75k	>75k-100k
WT vs Grb10$^{m/+}$	ns	ns	ns	ns
WT vs Dlk1$^{+/p}$	ns	ns	ns	ns
WT vs Grb10$^{m/+}$/Dlk1$^{+/p}$	ns	ns	ns	ns
Grb10$^{m/+}$ vs Dlk1$^{+/p}$	ns	ns	ns	ns
Grb10$^{m/+}$ vs Grb10$^{m/+}$/Dlk1$^{+/p}$	ns	ns	ns	ns
Dlk1$^{+/p}$ vs Grb10$^{m/+}$/Dlk1$^{+/p}$	ns	ns	ns	ns
