# Supplementary material for: Antagonistic roles in fetal development and adult physiology for the oppositely imprinted Grb10 and Dlk1 genes
Source: BMC Biol. 2014 Dec 31;12:771. doi: 10.1186/s12915-014-0099-8 (PMC4280702; doi:10.1186/s12915-014-0099-8)
Supplement: Additional file 3: Figure S3. — Whole body and organ wet weight analysis of wild type, Dlk1 +/p , Grb10 m/+ and Grb10 m/+ /Dlk1 +/p neonates. A) Grb10 m/+ and Grb10 m/+ /Dlk1 +/p animals showed whole body overgrowth, whereas Dlk1 +/p mice were significantly growth retarded when compared to wild type and Dlk1 +/p mice. Note, this graph is the same as that shown in Figure 4A, reproduced here for convenience. B) Grb10 m/+ and Grb10 m/+ /Dlk1 +/p mice had overgrown brains when compared to wild type and Dlk1 +/p animals. C) Significant enlargement of the livers was noted in Grb10 m/+ and Grb10 m/+ /Dlk1 +/p mice compared to wild type and Dlk1 +/p animals. D) Significant overgrowth of kidneys was seen in Grb10 m/+ /Dlk1 +/p mice compared to Dlk1 +/p. E) Significant enlargement of the lungs was observed in Grb10 m/+ and Grb10 m/+ /Dlk1 +/p mice compared to wild type and Dlk1 +/p animals. F) Grb10 m/+ and Grb10 m/+ /Dlk1 +/p mice exhibited significantly enlarged hearts compared to wild type and Dlk1 +/p animals. G) Table summarising results of statistical analysis. All values represent means ± SEM, analysed using one way ANOVA with Tukey’s post-hoc analysis. WT n = 19, Dlk1 +/p n = 36, Grb10 m/+ n = 23, Grb10 m/+ /Dlk1 +/p n = 22; * p < 0.05; *** P <0.001. [file 12915_2014_99_MOESM3_ESM.pdf]

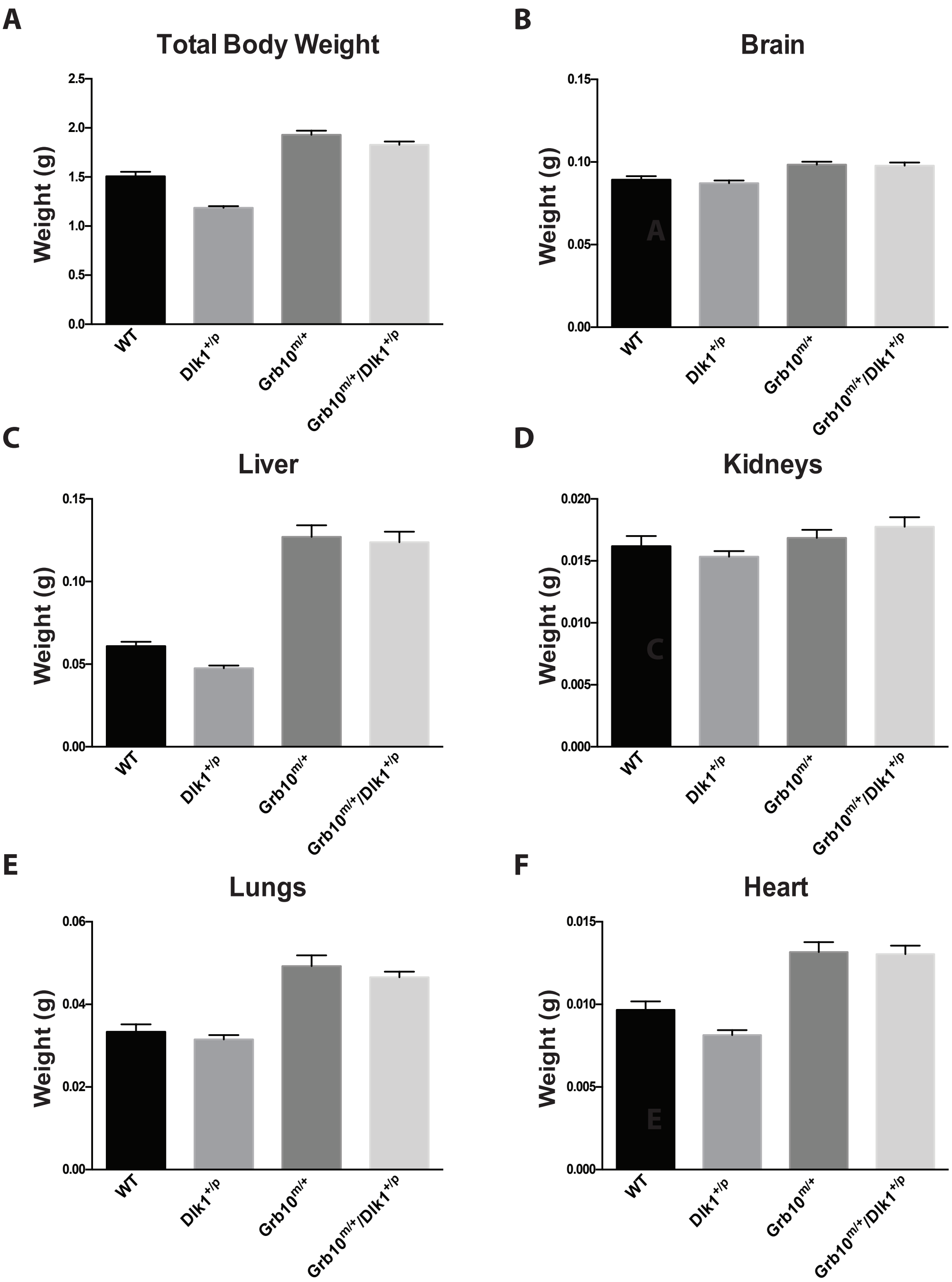

G

	Total body weight	Brain	Liver	Kidneys	Lungs	Heart
WT vs $Grb10^{m/+}$	***	*	***	ns	***	***
WT vs $Dlk1^{+/p}$	***	ns	ns	ns	ns	ns
WT vs $Grb10^{m/+}/Dlk1^{+/p}$	***	*	***	ns	***	***
$Grb10^{m/+}$ *vs* $Dlk1^{+/p}$	***	***	***	ns	***	***
$Grb10^{m/+}$ *vs* $Grb10^{m/+}/Dlk1^{+/p}$	ns	ns	ns	ns	ns	ns
$Dlk1^{+/p}$ *vs* $Grb10^{m/+}/Dlk1^{+/p}$	***	***	***	*	***	***
