# Supplementary figures and images for: Antagonistic roles in fetal development and adult physiology for the oppositely imprinted Grb10 and Dlk1 genes
Source: BMC Biol. 2014 Dec 31;12:771. doi: 10.1186/s12915-014-0099-8 (PMC4280702; doi:10.1186/s12915-014-0099-8)

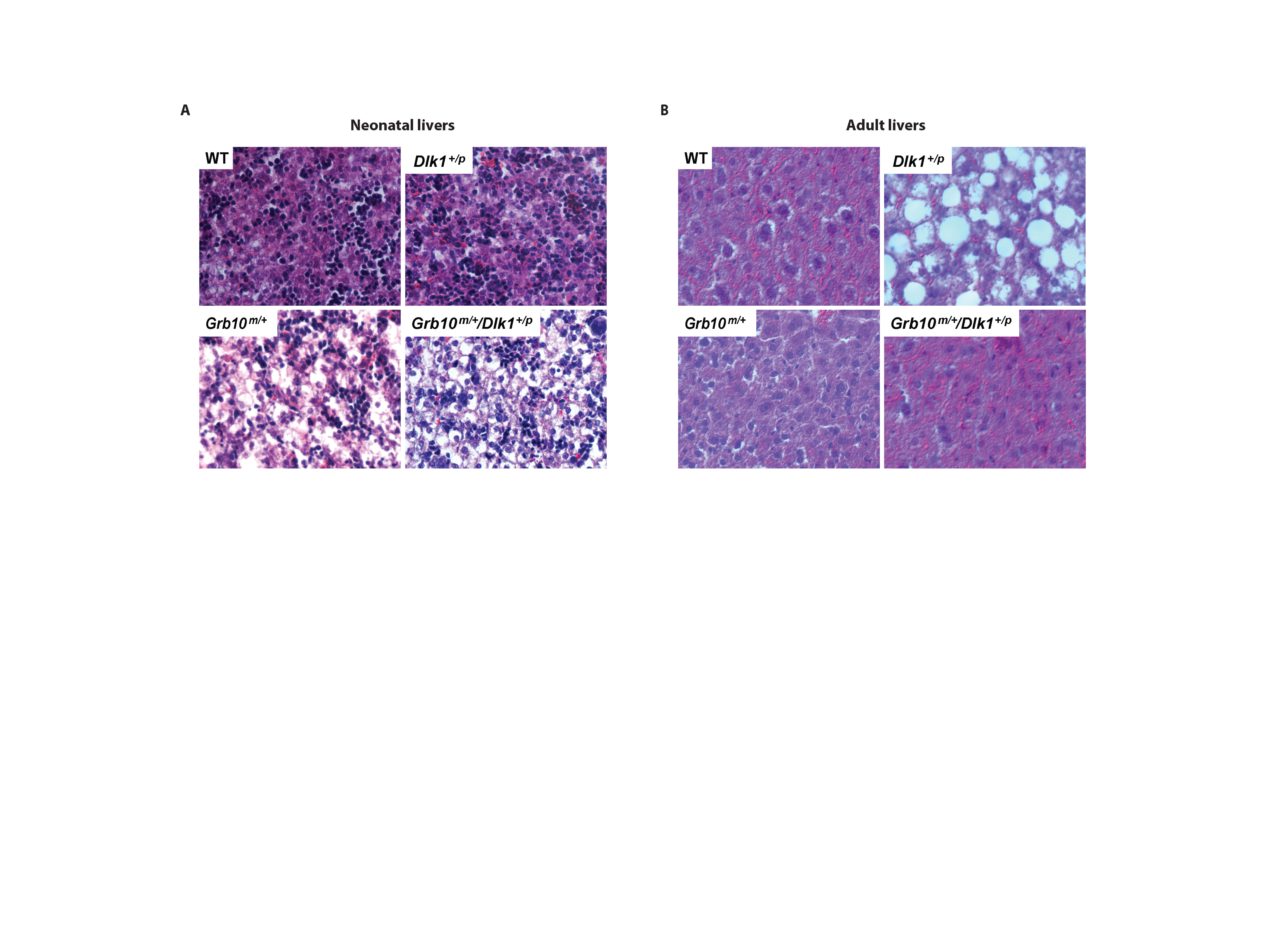

Supplement: Additional file 4: Figure S4. — Histology of neonatal and adult livers stained with H & E. A) Histological examination of neonatal livers stained with H & E revealed that Dlk1 +/p sections were indistinguishable from wild type, but Grb10 m/+ and Grb10 m/+ /Dlk1 +/p livers showed the presence of abundant white, round spaces (WT n = 5, Dlk1 +/p n = 6, Grb10 m/+ n = 4 and Grb10 m/+ /Dlk1 +/p n = 5). B) There were no differences in wild type, Grb10 m/+ and Grb10 m/+ /Dlk1 +/p adult livers (from males at three months of age); however, the presence of large and abundant white, round spaces was noted in Dlk1 +/p livers (n = 5 for each genotype). Presented images (in A and B) show representative sections for each of the analysed genotypes. [file 12915_2014_99_MOESM4_ESM.png]

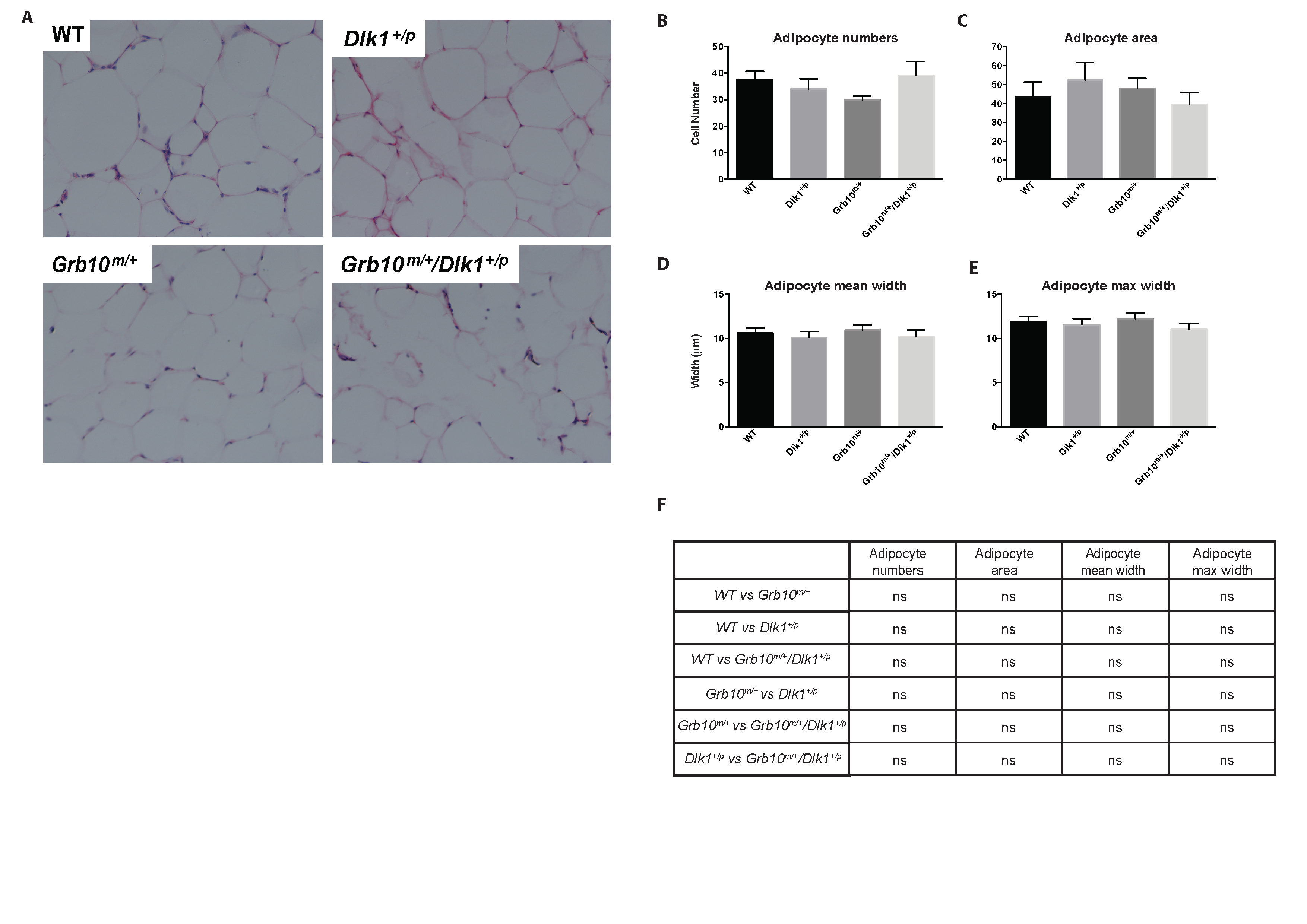

Supplement: Additional file 6: Figure S6. — Analysis of white adipose tissue sections from three-month-old mice. A) H & E stained sections of white adipose tissue. No obvious differences were noted between any of the analysed genotypes. Images are representative sections viewed at 100x magnification for each of the analysed genotypes, WT n = 6, Dlk1 +/p =5, Grb10 m/+ n = 6 and Grb10 m/+ /Dlk1 +/p n = 6. B-E) Morphometric analysis was carried out on images captured at 200x magnification, used to analyse adipocyte cell numbers (B) and cell areas (C), as well as mean (D) and maximum cell widths (E). F) Table summarising results of statistical analysis. All values represent means ± SEM and have been subject to one way ANOVA with post hoc Tukey’s analysis. WT n = 5, Dlk1 +/p n = 5, Grb10 m/+ n = 5 and Grb10 m/+ /Dlk1 +/p n = 5. No significant differences (ns; P >0.05) were found for any of the analysed parameters. [file 12915_2014_99_MOESM6_ESM.png]

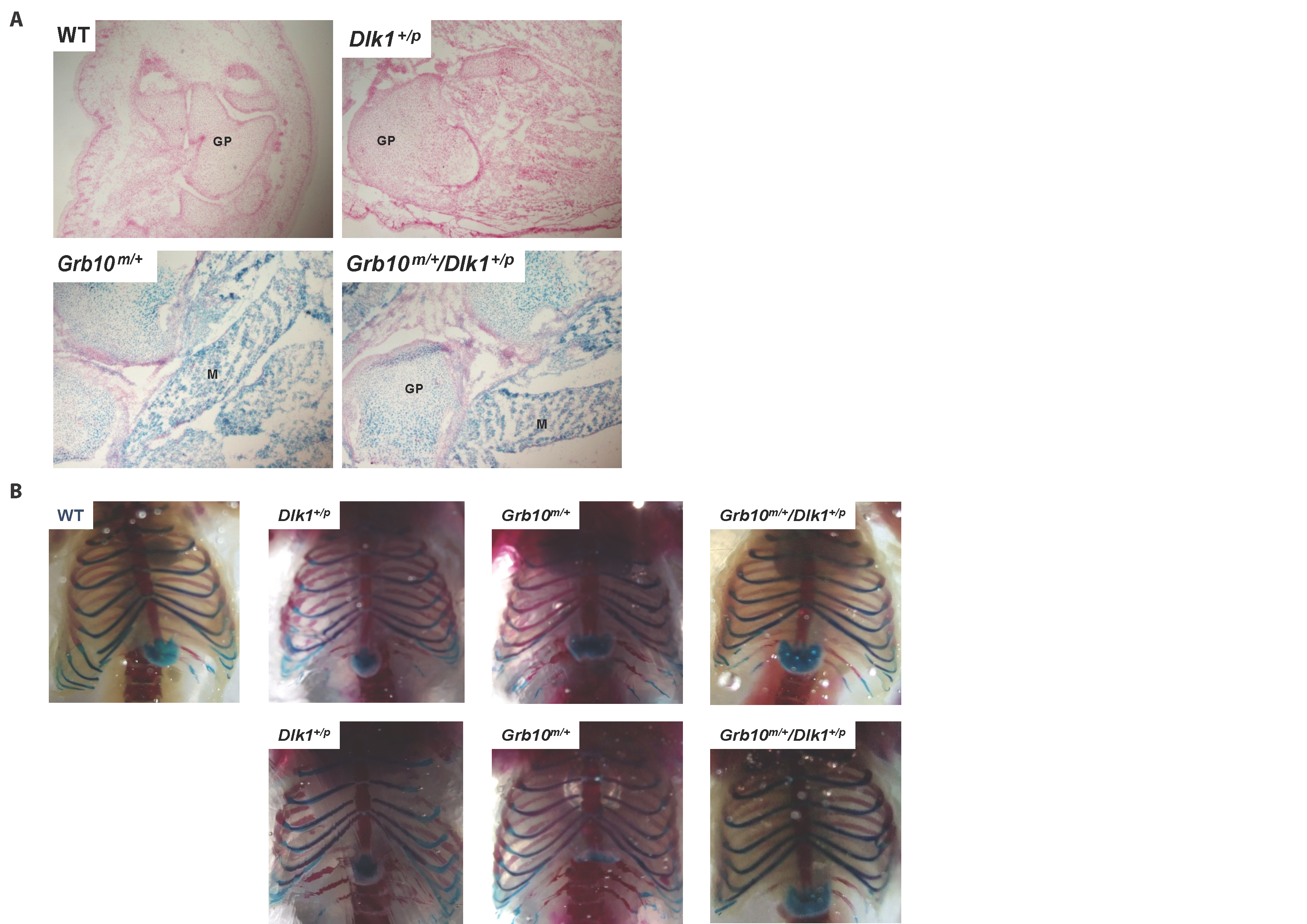

Supplement: Additional file 8: Figure S8. — Skeletal analyses. A) In sections of neonatal wild type, Grb10 m/+, Dlk1 +/p and Grb10 m/+ /Dlk1 +/p femurs LacZ (blue) staining was detected in Grb10 m/+ and Grb10 m/+ /Dlk1 +/p mice, specifically in growth plates and muscle tissue adjacent to the long bones, but not in wild type and Dlk1 +/p mice, which do not carry an integrated LacZ reporter gene. The images show the distal growth plate (GP) of the femur, with associated muscle (M) and in some cases the knee joint and adjacent growth plate of the tibia. Presented images (400x magnification) show representative sections for each of the analysed genotypes. WT n = 4, Dlk1 +/p n = 4, Grb10 m/+ n = 6 and Grb10 m/+ /Dlk1 +/p n = 3. B) Alcian Blue (cartilage) and Alizarin Red (bone) staining of skeletons from mice at two weeks of age revealed no obvious differences, including in the ribs and sternum previously reported to be malformed in Dlk1 knockout mice (Moon et al. [49]). [file 12915_2014_99_MOESM8_ESM.png]

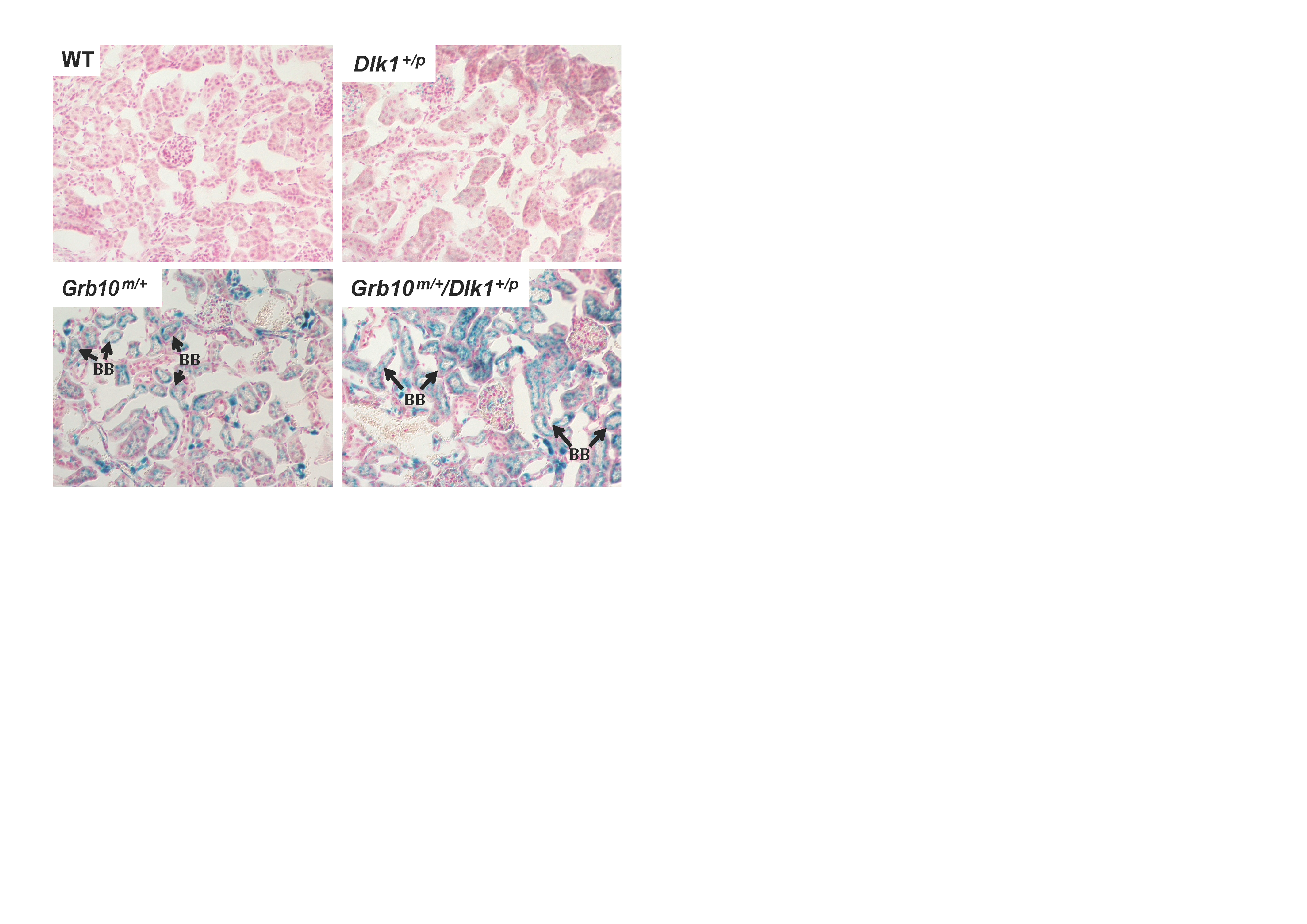

Supplement: Additional file 9: Figure S9. — Histology of adult kidney. In kidney sections from three-month-old males LacZ (blue) staining was observed in proximal tubules (characterised by distinctive brush border cells; (BB) of Grb10 m/+ and Grb10 m/+ /Dlk1 +/p, but not WT or Dlk1 +/p, mice. Presented images show representative sections for each of the analysed genotypes, counterstained with nuclear fast red (200x magnification). WT n = 4, Dlk1 +/p n = 3, Grb10 m/+ n = 3 and Grb10 m/+ /Dlk1 +/p n = 3. [file 12915_2014_99_MOESM9_ESM.png]
