# Supplementary material for: Antagonistic roles in fetal development and adult physiology for the oppositely imprinted Grb10 and Dlk1 genes
Source: BMC Biol. 2014 Dec 31;12:771. doi: 10.1186/s12915-014-0099-8 (PMC4280702; doi:10.1186/s12915-014-0099-8)
Supplement: Additional file 5: Figure S5. — DXA analysis of adult wild type, Grb10 m/+, Dlk1 +/p and Grb10 m/+ /Dlk1 +/p female mice. Carcasses of female animals three- to nine-months-old were analysed by DXA. A) No differences were found in bone mineral density (BMD). B) Bone mineral content was significantly reduced in Dlk1 +/p mice in comparison to wild type. C) Total lean tissue mass was significantly elevated in Grb10 m/+ animals when compared to Dlk1 +/p D) No changes were seen in total fat tissue content. E) Lean mass as a percentage of total body mass was significantly increased in Grb10 m/+ mice in comparison to Dlk1 +/p mice. F) Fat mass as a percentage of total body mass was significantly reduced in Grb10 m/+ mice when compared to Dlk1 +/p. G) Table summarising results of statistical analysis. All values represent means ± SEM and have been subject to one way ANOVA with post hoc Tukey’s analysis. WT n = 13, Dlk1 +/p n = 12, Grb10 m/+ n = 12 and Grb10 m/+ /Dlk1 +/p n = 12; * P <0.05; ** P <0.01. [file 12915_2014_99_MOESM5_ESM.pdf]

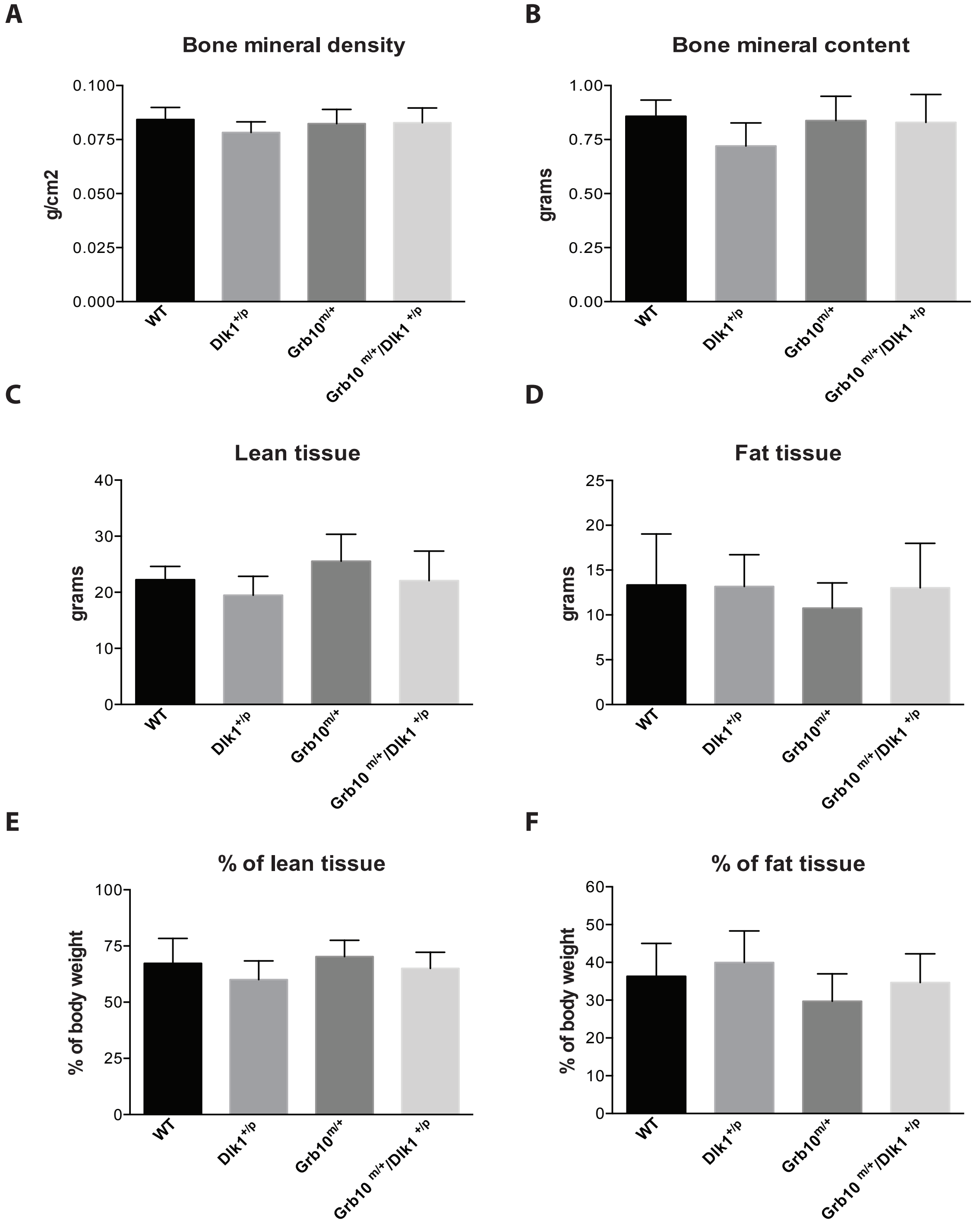

G

	BMD	BMC	Lean tissue	Fat tissue	% lean tissue	% fat tissue
WT vs $Grb10^{m/+}$	ns	ns	ns	ns	ns	ns
WT vs $Dlk1^{+/p}$	ns	*	ns	ns	ns	ns
WT vs $Grb10^{m/+}/Dlk1^{+/p}$	ns	ns	ns	ns	ns	ns
$Grb10^{m/+}$ *vs* $Dlk1^{+/p}$	ns	ns	**	ns	*	*
$Grb10^{m/+}$ *vs* $Grb10^{m/+}/Dlk1^{+/p}$	ns	ns	ns	ns	ns	ns
$Dlk1^{+/p}$ *vs* $Grb10^{m/+}/Dlk1^{+/p}$	ns	ns	ns	ns	ns	ns
