# Supplementary material for: Antagonistic roles in fetal development and adult physiology for the oppositely imprinted Grb10 and Dlk1 genes
Source: BMC Biol. 2014 Dec 31;12:771. doi: 10.1186/s12915-014-0099-8 (PMC4280702; doi:10.1186/s12915-014-0099-8)
Supplement: Additional file 7: Figure S7. — Analysis of food consumption. Food intake was monitored over a period of two weeks. Total consumption is shown for males (A) and females (B) and food consumption rates have been calculated as food consumed per gram of animal body weight per day to the power of 2/3 (to take account of basal metabolic rate as a function of body mass [83]), again for males (C) and females (D). E) Table summarising results of statistical analysis. All values represent means ± SEM and have been subject to one way ANOVA with post hoc Tukey’s analysis. Males: WT n = 14, Dlk1 +/p =12, Grb10 m/+ n = 12 and Grb10 m/+ /Dlk1 +/p n = 13; females: WT n = 13, Dlk1 +/p n = 12, Grb10 m/+ n = 12 and Grb10 m/+ /Dlk1 +/p n = 12. No significant differences (ns, P >0.05) were found in either the total amount of food consumed by males and females or in the rate of food consumption. [file 12915_2014_99_MOESM7_ESM.pdf]

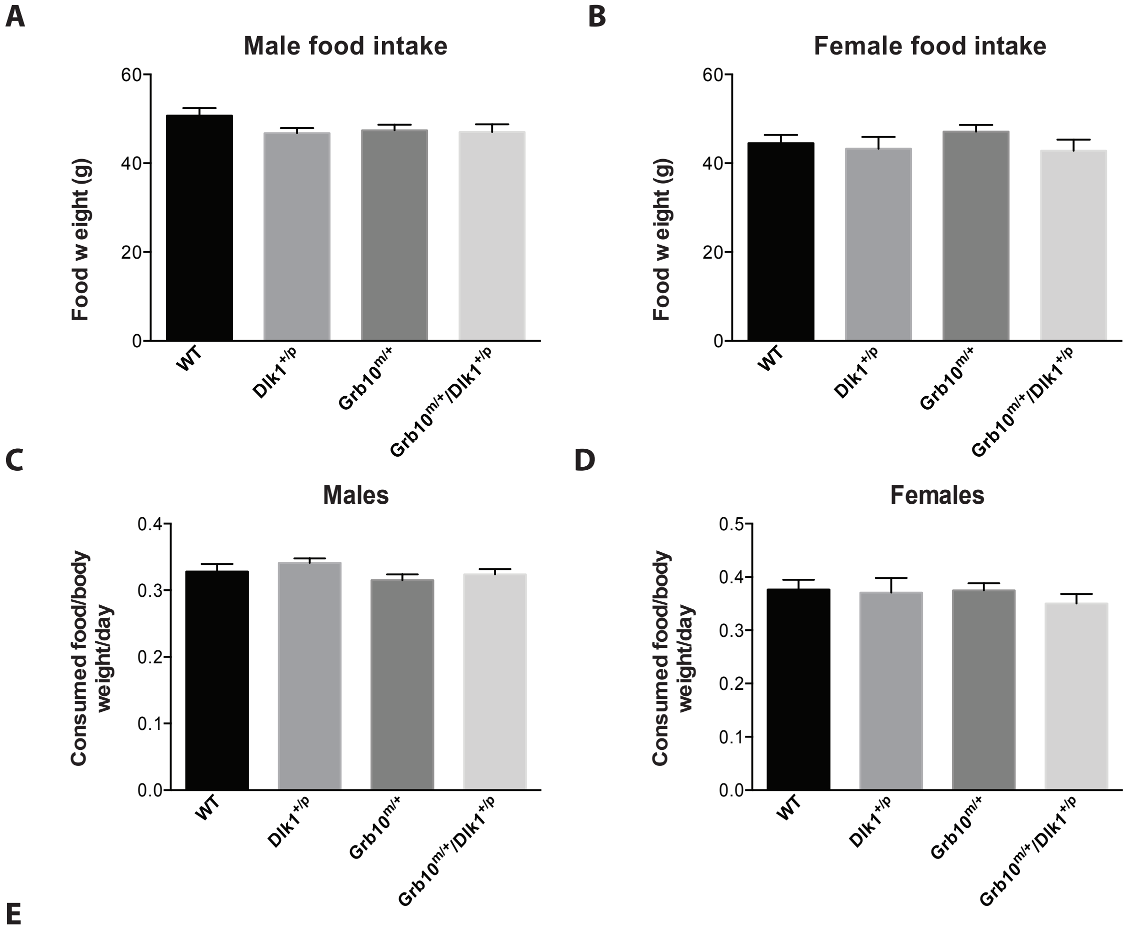

E

	Food males	Food females	Consumption rate males	Consumption rate females
WT vs $Grb10^{m/+}$	ns	ns	ns	ns
WT vs $Dlk1^{+/p}$	ns	ns	ns	ns
WT vs $Grb10^{m/+}/Dlk1^{+/p}$	ns	ns	ns	ns
$Grb10^{m/+}$ *vs* $Dlk1^{+/p}$	ns	ns	ns	ns
$Grb10^{m/+}$ *vs* $Grb10^{m/+}/Dlk1^{+/p}$	ns	ns	ns	ns
$Dlk1^{+/p}$ *vs* $Grb10^{m/+}/Dlk1^{+/p}$	ns	ns	ns	ns
